# Comparison of neurodegenerative types using different brain MRI analysis metrics in older adults with normal cognition, mild cognitive impairment, and Alzheimer’s dementia

**DOI:** 10.1371/journal.pone.0220739

**Published:** 2019-08-01

**Authors:** Myungwon Choi, HyunChul Youn, Daegyeom Kim, Suji Lee, Sangil Suh, Joon-Kyung Seong, Hyun-Ghang Jeong, Cheol E. Han

**Affiliations:** 1 Department of Electronics and Information Engineering, Korea University, Sejong, Republic of Korea; 2 Department of Psychiatry, Korea University Guro Hospital, Korea University College of Medicine, Seoul, Republic of Korea; 3 Department of Biomedical Sciences, Korea University Graduate School, Seoul, Republic of Korea; 4 Department of Radiology, Korea University Guro Hospital, Korea University College of Medicine, Seoul, Republic of Korea; 5 Department of Bio-convergence Engineering, Korea University, Seoul, Republic of Korea; 6 Korea University Research Institute of Mental Health, Seoul, Republic of Korea; Nathan S Kline Institute, UNITED STATES

## Abstract

Several metrics of analysis of magnetic resonance imaging (MRI) have been used to assess Alzheimer’s disease (AD)-related neurodegeneration. We compared four structural brain MRI analysis metrics, cortical thickness, volume, surface area, and local gyrification index (LGI), in different stages of AD-related cognitive decline. Participants with normal cognition, mild cognitive impairment, and AD were included (34 participants per group). All undertook the Consortium to Establish a Registry for Alzheimer’s Disease (CERAD) battery of neuropsychological tests and brain MRI scanning. We analyzed associations between morphometric measures and CERAD total/ Mini Mental State Examination (MMSE) scores for the regions of interest (ROIs), identifying three types of curves: U-shaped, inverted U-shaped, and linear. Cortical thickness and volume analyses showed linear types in most of the significant ROIs. Significant ROIs for the cortical thickness analysis were located in the temporal and limbic lobes, whereas those for volume and surface area were distributed over more diffuse areas of the brain. LGI analysis showed few significant ROIs. CERAD total scores were more sensitive to early changes of cortical structures than MMSE scores. Cortical thickness analysis may be preferable in assessing brain structural MRI changes during AD-related cognitive decline, whereas LGI analysis may have limited capability to reflect the cognitive decrease. Our findings may provide a reference for future studies and help to establish optimal analytical approaches to brain structural MRI in neurodegenerative diseases.

## Introduction

Several computerized methodologies for the analysis of structural magnetic resonance imaging (MRI) analysis have been used to assess Alzheimer’s disease (AD)-related neurodegeneration. Brain atrophy is a well-known marker of neurodegeneration reflecting pathological changes in AD and is associated with a reduction in volume that can be observed by brain structural MRI [1]. A recent surface-based analysis reported that cortical thinning of various regions is associated with both normal aging and AD-related cognitive decline [2]. Querbes et al. showed that normalized cortical thickness indices computed in the medial temporal, lateral temporal, and posterior cingulate were related to the early diagnosis of AD [3]. Bakkour et al. also found that progressors to AD showed temporal and parietal thinning [4]. Such cortical thinning reflects neurodegenerative processes such as cell loss associated with AD-related pathology [2]. Another interesting measurement from surface-based analysis is the quantified gyrification index. The human brain cortex consists of highly folded sulci and gyri. These folds in the cortex can be widened due to cortical atrophy associated with AD-related neurodegeneration [5]. The brain’s cortical folding geometry can be quantified with the local gyrification index (LGI) [6], which can measure the sulcal widening induced by brain atrophy. Ruiz de Miras et al. conducted LGI analysis in AD patients, and found that LGI was reduced in posterior-medial temporal lobe compared to healthy controls [2].

Despite several previous studies, the strengths and weaknesses of each metric of analysis of structural brain MRI in assessing neurodegeneration, including diagnosis, stage, severity, and prognosis of the diseases are not fully understood. They are related and complementary to the understanding of the effects of brain atrophy on the structural changes of the cortices. For example, brain atrophy not only leads to cortical thinning, but also to a reduction of the surface area. The surface area has been known to decrease during the aging process, but few studies have assessed the changes of surface area in individuals with cognitive decline [7, 8]. Reduced surface area is one of the major factors contributing to volume reduction, which is associated to AD [9, 10]. Therefore, potential associations between surface area and AD may be expected. Considering that brain structural MRI is the marker of AD-related neurodegeneration [1], comparison of AD-related neurodegenerative types using different brain structural analysis metrics may provide a reference to determine the appropriate analysis metrics for the various stages of cognitive decline, and enhance our understanding of the characteristics of each metric.

We compared four structural brain MRI analysis metrics in the different stages of AD-related cognitive decline. Cortical thickness, volume, surface area, and LGI analyses were conducted in older adults with normal cognition (NC), mild cognitive impairment (MCI), and AD. To compare these four metrics, we analyzed the relationship between the structural measurements and the severity of cognitive dysfunction in 68 regions-of-interest (ROIs) using Mini Mental State Examination (MMSE) and Consortium to Establish a Registry for Alzheimer’s Disease (CERAD) total scores.

## Materials and methods

### Participants

This study recruited an equal number of NC, MCI, and AD participants (34 participants per group) from the Guro-gu Center for Dementia and Korea University Guro Hospital. This study used the criteria for probable AD established by the National Institute of Neurological and Communicative Disorders and Stroke and the Alzheimer’s Disease and Related Disorders Association to classify AD patients [11]. MCI and NC participants were age-and-gender matched with AD participants. The diagnosis of MCI was based on the revised diagnostic criteria for MCI proposed by the International Working Group on MCI [12]. NC participants had no previous history of psychiatric or neurologic disorders. Participants with other neurological impairments (e.g., encephalosclerosis, epilepsy, or traumatic brain injury) or with claustrophobia, substance abuse disorders, and/or with cardiac pacemakers, which can affect the MRI results, were excluded from the study. All participants with NC, MCI, and AD underwent MRI testing, answered demographic questionnaires and underwent a battery of neuropsychological tests. All the study subjects provided written informed consent prior to participation. Capacity to provide consent was determined by group classification. All written informed consents of NC participants were obtained directly from the participants. In MCI and AD groups, written informed consents were obtained from both the participants and legal guardian. This study protocol and consent procedure were approved by the Institutional Review Board at Korea University Guro Hospital (2016GR0003/2016GR0200).

### MRI acquisition

All participants underwent structural MRI examination at the Brain Imaging Center of Korea University. Three-dimensional structural MRI scans were acquired from a 3.0 T Siemens Trio whole-body imaging system (Siemens Healthineers, Erlangen, Germany), using a T1-weighted magnetization-prepared rapid acquisition gradient-echo (MP-RAGE 1900 ms repetition time, 2.6 ms echo time, 900 ms inversion time, 220 mm field of view, 256 × 256 matrix size, 176 coronal slices without gap, 0.86 × 0.86 × 1 mm^3^ voxels, 9° flip angle, number of excitations = 1).

### Image processing and cortical measurements

We analyzed four cortical measurements using the FreeSurfer software (v5.3.0): Cortical thickness, volume, surface area, and LGI. FreeSurfer is a surface-based analysis tool providing various cortical measurements. The technical details are described in the original publications [13–16]. Here, we briefly explain the overall procedure and the elements we used. From T1-weighted MR images, it extracts pial and white surfaces through its semi-automated pipeline, where the pial surface corresponds to the boundary between the gray matter and the cerebrospinal fluid, and the white surface marks the boundary between white and gray matter. The cortical surfaces are tessellations of those boundaries with small triangles, and thus consist of vertices, and are parceled into 68 anatomically distinct ROIs based on the gyral and sulcal structures [6, 17].

We used each value of cortical thickness, cortical gray matter volume, cortical surface area, and LGI over the 68 predefined ROIs. These cortical measurements are computed by the automated pipeline of FreeSurfer at the each vertex of the small triangles on the surfaces [18, 19]. The cortical thickness at each vertex estimates the shortest Euclidean distance from the white surface to the pial surface [15]. The surface area at each vertex evaluates the average of the area of the triangles touching that vertex on the pial surface [20]. The cortical volume is calculated by the production of the surface area and the thickness at each vertex [21]. The LGI quantifies the amount of cortex buried within the sulcal folds as compared with the amount of cortex on the visible outer cortex in the ROIs. In this study, we used the FreeSurfer implementation, as previously described [6, 16]. Briefly, a large or small gyrification index indicates highly folded or smooth cortex, respectively; it usually ranges from 1 to 5, where 1 indicates a flat pial surface. After obtaining each cortical measurement value at all vertex, we averaged each measurement over the 68 ROIs.

### Demographic and cognitive measures

Data on age, sex, and educational level was acquired for all participants. We conducted the Korean version of the CERAD neuropsychological battery of clinical tests to evaluate the cognitive functions of the participants [22, 23]. The CERAD total score was obtained by summing the scores of the individual CERAD subdomains, except the MMSE, constructional recall, and trail making tests, with a cap of 24 placed on verbal fluency [24]. The MMSE is a widely used measure of cognitive function for screening and clinical evaluation of patients with AD [25]: This study adopted the Korean version of MMSE in the CERAD assessment packet [26].

### Statistical analysis

Differences between the NC, MCI, and AD groups in demographic and cognitive measures were analyzed using Predictive Analytics Software version 18.0 for Windows. We used analysis of variance with Bonferroni post hoc tests for continuous variables, and chi squared tests for categorical variables.

We analyzed the effects of AD-related cognitive decline on each cortical measurement using general linear models (GLMs). We established three types of functional dependence: U-shaped, inverted U-shaped, and linear. These types are defined by two types of GLM equations. The first equation is quadratic and contains second-order regression coefficient term, *β*_*1*_, associated with the MMSE/CERAD total score:

$$y=\beta_{0}+\beta_{1}∙{score}^{2}+\beta_{2}∙score+\beta_{3}∙age+\beta_{4}∙sex+\beta_{5}∙education+\beta_{6}∙ICV$$

where *y* is the cortical measure, and *β*_*0*_, *β*_*2*_, *β*_*3*_, *β*_*4*_, *β*_*5*_, and *β*_*6*_ are the regression coefficients for each term: bias, MMSE/CERAD total score, age, sex, education level, and intracranial volume (ICV) for each subject. The first two types (U-shaped and inverted U-shaped) were determined when the second-order term in GLM was significant according to the analysis of covariance (ANCOVA) test. We also assessed the goodness-of-fit using the R^2^, that is the ratio between the regression sum of squares and the total sum of square obtained from ANCOVA. A higher R^2^ indicates a better fit of the regression line to the data: When R^2^ is greater than 0.26, the level of fitting is considered substantial [27]. Thus, we included the results for which R^2^ was greater than 0.26.

For each ROI, we applied the GLM described above. When the goodness-of-fit was greater than 0.26 and the coefficient of the second-order term, *β*_*1*_, was significant, we defined U-shaped and inverted U-shaped types, respectively for positive and negative values of *β*_*1*_.

Otherwise, if the *β*_*1*_ coefficient was not significant or the goodness of fit was too low, we used the alternative GLM model without the second-order term:

$$y=\alpha_{0}+\alpha_{1}∙score+\alpha_{2}∙age+\alpha_{3}∙sex+\alpha_{4}∙education+\alpha_{5}∙ICV$$

where *y* is the cortical measure, and α_*0*_, α_*2*_, α_*3*_, α_*4*_, and α_*5*_ correspond to the regression coefficients for each term: bias, age, sex, education level, and ICV, respectively, for each subject. Similarly, to the other two types, when the coefficient of first-order term, α_*1*_, was significant and the goodness-of-fit was over 0.26, we defined the “linear type”. We illustrated this procedure as a flowchart in S1 Fig.

We used the F test for estimating significance levels of the coefficients *β*_1_ and *α*_1_. The F test decides how significantly a certain term improves the explainability of the model using the F-distribution. To perform the test, first, we fit two models: the full model and a nested model that does not contain the term. Then the F statistics is computed based on the residual sum-of-squares and degree-of-freedoms of two models, and the significance level is estimated using the F-distribution. For example, to compute the significance model of the coefficient *β*_1_, the first equation is the full model, while the second equation is the nested model, since the former contains the term with *β*_1_. We used the linstats 2006b toolbox (http://www.mathworks.com/matlabcentral/fileexchange/13493-linstats-2006b) for fitting the GLM and estimating the significance levels. We used our in-house MATLAB codes for all other analyses and visualization on Matlab 8.1 (64bit version, R2013a, Mathworks, Natick, USA).

### Three types of descending graphs

We classified the relationship between structural measures and the severity of cognitive dysfunction into 3 types as follows: 1) U-shaped type, 2) linear type and 3) inverted U-shaped type.

**Fig 1:**
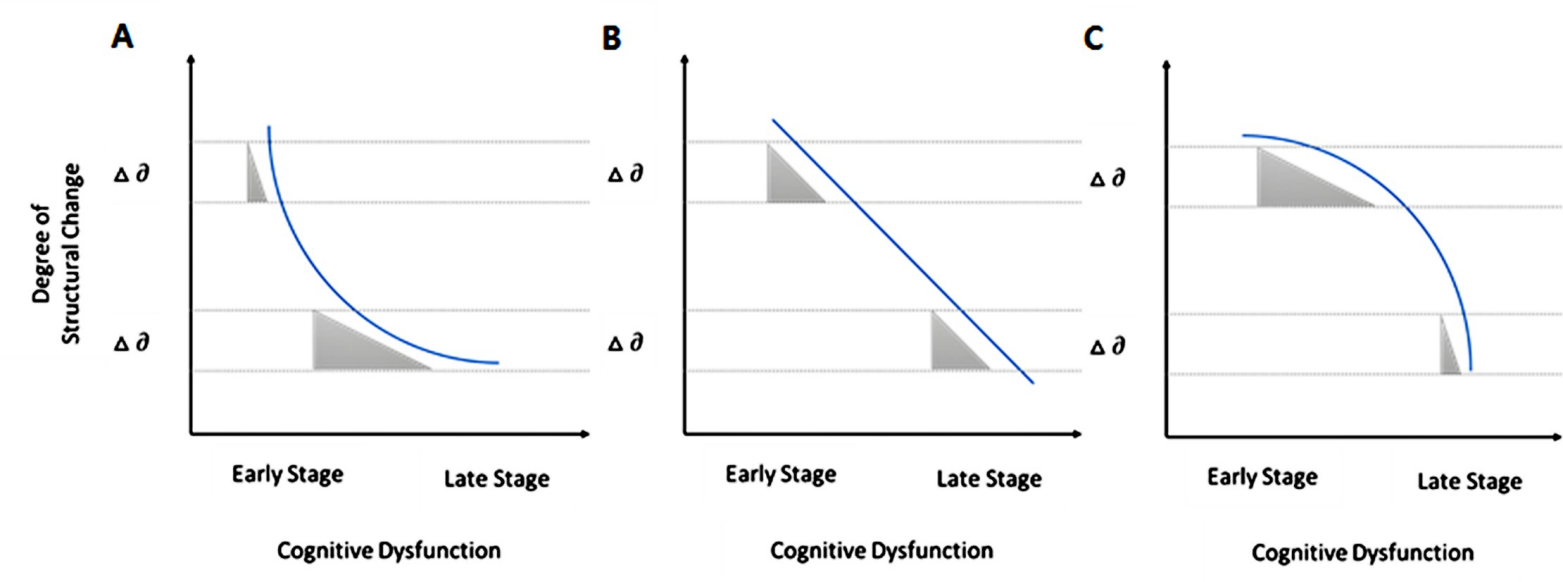
Schematic graphs of (A) U-shaped type, (B) linear type, and (C) inverted U-shaped type.

*U-shaped type*: The gradient of the structural measure as a function of cognitive decline is larger in the early than in the late stage of cognitive dysfunction (Fig 1A). This means that the analysis metric can reflect with high sensitivity cognitive impairment in the early stage of the disease.

*Linear type*: The gradient of the structural measure as a function of cognitive decline is stable from the early to the late stage (Fig 1B). Hence this analysis metric can reflect cognitive impairment with comparable sensitivity over all disease stages.

*Inverted U-shaped type*: The gradient of the structural measure as a function of cognitive decline is smaller in the early than in the late stage of cognitive dysfunction (Fig 1C). This analysis metric can reflect with high sensitivity cognitive impairment in the late stage of the disease.

## Results

Table 1 shows the basic characteristics and cognitive measures of the NC, MCI, and AD groups. The mean ages of these groups were 72.85 (SD = 6.09), 74.68 (SD = 10.05), and 76.56 (SD = 7.50), respectively. More females than males participated in all three groups. As expected, the three groups significantly differed in MMSE and CERAD test scores.

**Table 1:** Basic characteristics and cognitive measures of NC, MCI, and AD groups.

	NC (n = 34)	MCI (n = 34)	AD (n = 34)	ANOVA *p* value	Bonferroni post hoc test “*p* value”		
					NC vs. MCI	NC vs. AD	MCI vs. AD
Age (years)	72.85±6.09	74.68±10.05	76.56±7.50	0.170	1.000	0.181	1.000
Sex (male/female)	8/26	7/27	6/28	0.835^a^			
Education (years)	6.32±5.46	5.82±4.80	5.29±4.83	0.702	1.000	1.000	1.000
ICV (x10^5^mm^3^)	13.25±1.28	13.24±1.11	13.20±1.12	0.986	1.000	1.000	1.000
MMSE	24.42±4.45	20.00±5.40	12.82±4.77	<0.001**	0.001**	<0.001**	<0.001**
CERAD							
Constructional praxis	8.84±1.83	7.82±2.24	6.71±2.83	0.002**	0.245	0.001**	0.167
Word list memory	15.25±4.81	8.76±3.77	5.82±4.39	<0.001**	<0.001**	<0.001**	0.020*
Word list recall	5.41±1.81	2.09±2.13	0.56±0.75	<0.001**	<0.001**	<0.001**	0.001**
Word list recognition	9.03±1.06	5.48±2.90	3.03±2.69	<0.001**	<0.001**	<0.001**	<0.001**
Verbal fluency	12.31±5.13	10.48±3.78	6.45±3.76	<0.001**	0.262	<0.001**	0.001**
Boston naming test	10.75±2.69	7.97±3.22	5.56±2.56	<0.001**	<0.001**	<0.001**	0.002**
Total score	61.16±10.50	42.61±12.75	28.45±12.04	<0.001**	<0.001**	<0.001**	<0.001**

*P* values were calculated using ANOVA with Bonferroni post hoc tests and chi-squared test.

* *P* < 0.05

** *P* < 0.01. NC, Normal Cognition; MCI, Mild Cognitive Impairment; AD, Alzheimer’s disease; ANOVA, analysis of variance; ICV, Intracranial Volume; MMSE, Mini Mental State Examination; CERAD, Consortium to Establish a Registry for Alzheimer’s Disease.

**Fig 2:**
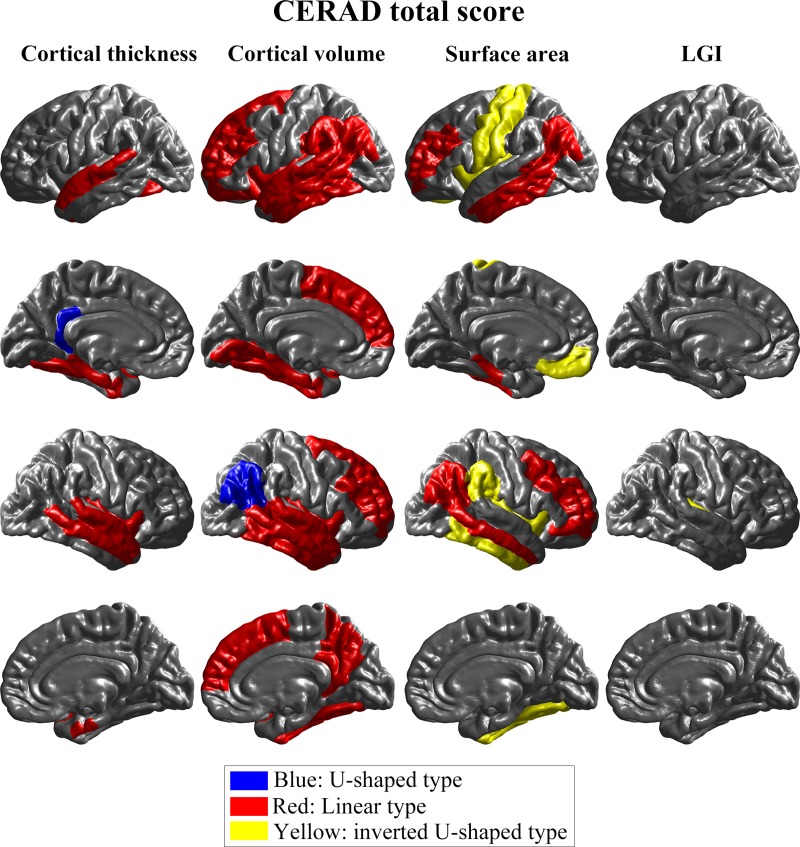
Comparison of descending graph types between cortical volume, thickness, surface area, and LGI in CERAD total score. The first and second rows show the left hemisphere from a lateral and medial view, respectively. Similarly, the third and fourth rows represent the right hemisphere from a lateral and medial view. The blue regions represent the U-shaped type and indicate sensitivity to cognitive impairment in the early stages of the Alzheimer disease. The red regions represent the linear type, showing that the ROI may be continuously damaged as the disease progresses. The yellow regions represent the inverted U-shaped type, meaning that cognitive decline is especially sensitive at the last stages. *LGI* local gyrification index.

Table 2 and Fig 2 show the types of descending graphs analyzed using four structural brain MRI analysis metrics, and CERAD total scores, in 68 ROIs. In the cortical thickness analysis, linear types were shown in the entorhinal, left fusiform, right middle temporal, left parahippocampal, superior temporal, and right insula regions, while the left isthmus cingulate region showed the U-shaped type. Volume analysis showed linear types in the banks of superior temporal sulcus, left entorhinal, fusiform, left inferior parietal, inferior temporal, right isthmus cingulate, left lateral orbitofrontal, left lingual, middle temporal, left parahippocampal, right precuneus, rostral middle frontal, superior frontal, superior temporal, left supramarginal, and insula regions. Only the U-shaped type was represented, in volume analysis, in the right inferior parietal region. Surface area analysis produced inverted U-shaped types in the right fusiform, right inferior temporal, left medial orbitofrontal, left postcentral, left precentral, right supramarginal, and insula regions, while linear types were shown in the left banks of superior temporal sulcus, right caudal middle frontal, inferior parietal, left inferior temporal, middle temporal, left parahippocampal, right pars orbitalis, and rostral middle frontal regions. In contrast, LGI analysis showed significant result in only one ROI, the left transverse temporal region, where it showed an inverted U-shaped type of descending graph. Descending graph types of the 68 ROIs over the CERAD total scores are displayed on the standard brain template in Fig 2. In summary, linear types in the cortical thickness analysis were mainly found in the temporal and limbic lobe. Volume analysis showed also mostly linear types in various ROIs. Surface area analysis produced linear and inverted U-shaped types in various ROIs, while LGI analysis showed only one significant ROI of the inverted U-shape type. The detailed results of statistical analyses can be found in S1–S8 Tables.

**Table 2:** Types of descending graph analyzed using four structural brain MRI analyses metrics using CERAD total scores in 68 ROIs.

	ROI names	T_lh	T_rh	V_lh	V_rh	SA_lh	SA_rh	LGI_lh	LGI_rh
1	bankssts			linear	linear	linear			
2	caudalanteriorcingulate								
3	caudalmiddlefrontal						linear		
4	cuneus								
5	entorhinal	linear	linear	linear					
6	fusiform	linear		linear	linear		inverted U		
7	inferiorparietal			linear	U	linear	linear		
8	inferiortemporal			linear	linear	linear	inverted U		
9	isthmuscingulate	U			linear				
10	lateraloccipital								
11	lateralorbitofrontal			linear					
12	lingual			linear					
13	medialorbitofrontal					inverted U			
14	middletemporal		linear	linear	linear	linear	linear		
15	parahippocampal	linear		linear		linear			
16	paracentral								
17	parsopercularis								
18	parsorbitalis						linear		
19	parstriangularis								
20	pericalcarine								
21	postcentral					inverted U			
22	posteriorcingulate								
23	precentral					inverted U			
24	precuneus				linear				
25	rostralanteriorcingulate								
26	rostralmiddlefrontal			linear	linear	linear	linear		
27	superiorfrontal			linear	linear				
28	superiorparietal								
29	superiortemporal	linear	linear	linear	linear				
30	supramarginal			linear			inverted U		
31	frontalpole								
32	temporalpole								
33	transversetemporal								inverted U
34	insula		linear	linear	linear	inverted U	inverted U		

*T_lh*, *T_rh* results using cortical thickness measurement in the left and right hemisphere, respectively, *V_lh*, *V_rh* results using volume in the left and right hemisphere, *S_lh*, *S_rh* results using surface area in left and right hemisphere, *LGI_lh*, *LGI_rh* results using local gyrification index of the left and right hemisphere. ROIs are defined using the anatomical Desikan–Killiany atlas with 68 parcels bilaterally.

**Fig 3:**
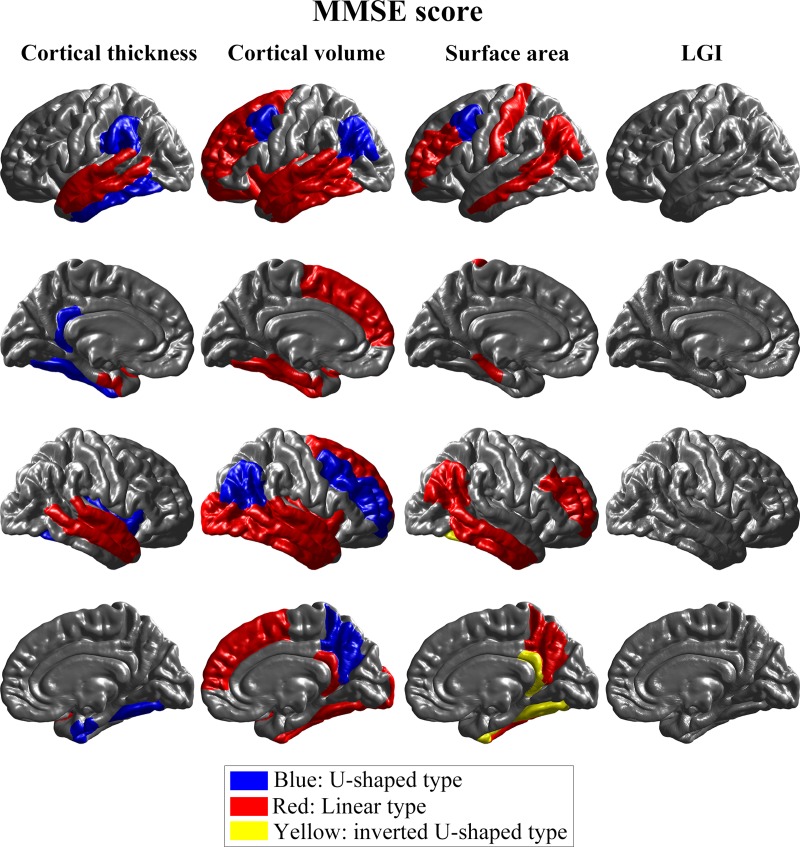
Comparison of descending graph types between cortical volume, thickness, surface area, and LGI in MMSE score. The first and second rows show the left hemisphere from a lateral and medial view, respectively. Similarly, the third and fourth rows represent the right hemisphere from a lateral and medial view. The blue regions represent the U-shaped type and indicates sensitivity to cognitive impairment in the early stages of Alzheimer disease. The red regions represent the linear type, showing that the ROI may be continuously damaged as the disease progresses. The yellow regions represent the inverted U-shaped type, meaning that cognitive decline is most sensitive at the last stages. *LGI* local gyrification index.

Table 3 and Fig 3 show the types of descending graphs determined using four structural brain MRI analysis metrics and MMSE scores in 68 ROIs. While the analysis of cortical thickness and volume showed significant results in the temporal and limbic lobes, the types were slightly different compared to those obtained with the CERAD total scores, in that they showed more U-shaped types and less linear types. Surface area analysis showed a relative increase in linear types, and decrease in inverted U-shaped types, compared to those obtained with the CERAD total scores. Only the caudal middle frontal regions showed a U-shaped type. LGI analysis using MMSE scores gave no significant ROIs. Descending graph types obtained from the MMSE scores are displayed on the standard brain template in Fig 3.

**Table 3:** Types of descending graph analyzed using four structural brain MRI analyses metrics using MMSE scores in 68 ROIs.

	ROI names	T_lh	T_rh	V_lh	V_rh	SA_lh	SA_rh	LGI_lh	LGI_rh
1	bankssts			linear	linear	linear			
2	caudalanteriorcingulate								
3	caudalmiddlefrontal			U	U	U			
4	cuneus								
5	entorhinal	linear	U	linear					
6	fusiform	U	U	linear	linear		inverted U		
7	inferiorparietal			U	U	linear	linear		
8	inferiortemporal	U		linear	linear		linear		
9	isthmuscingulate	U			linear		inverted U		
10	lateraloccipital				linear				
11	lateralorbitofrontal			linear					
12	lingual								
13	medialorbitofrontal								
14	middletemporal	linear	linear	linear	linear	linear	linear		
15	parahippocampal			linear		linear			
16	paracentral								
17	parsopercularis								
18	parsorbitalis								
19	parstriangularis								
20	pericalcarine								
21	postcentral					linear			
22	posteriorcingulate								
23	precentral								
24	precuneus				U		linear		
25	rostralanteriorcingulate								
26	rostralmiddlefrontal			linear	U	linear	linear		
27	superiorfrontal			linear	linear				
28	superiorparietal								
29	superiortemporal	linear	linear	linear	linear				
30	supramarginal	U							
31	frontalpole								
32	temporalpole								
33	transversetemporal								
34	insula		U	linear	linear				

*T_lh*, *T_rh* results using cortical thickness measurement in the left and right hemisphere respectively, *V_lh*, *V_rh* results using volume in the left and right hemisphere, *S_lh*, *S_rh* results using surface area in left and right hemisphere, *LGI_lh*, *LGI_rh* results of local gyrification index of the left and right hemisphere. ROIs are defined using the anatomical Desikan–Killiany atlas with 68 parcels bilaterally.

Comparing Figs 2 and 3, the results of the analyses using CERAD total scores have more linear and inverted U-shaped types, and fewer U-shaped types, than those based on MMSE scores. Specifically, there are respectively 43 and 9 linear and inverted U-shaped types in the CERAD total scores, and only 36 and 2 in the MMSE scores. Using the MMSE scores, the U-shaped types are found in 14 regions, but only in 2 using the CERAD total scores.

## Discussion

The type differences among cortical thickness, volume, surface area, and LGI represent our major findings. In the analysis using CERAD total scores, cortical thickness and volume showed linear types in most of the significant ROIs, whereas the surface area represented more significant ROIs of the inverted U-shaped type. The significant ROIs of cortical thickness analysis were found in the temporal and limbic lobes. In contrast, those of volume and surface area were distributed over more diffuse areas in the brain. There were few significant ROIs in LGI analysis. The analysis using MMSE scores showed that the distributions of significant ROIs were almost similar to those obtained using CERAD total scores. However, more U-shaped than linear types were found in cortical thickness and volume, and more linear than inverted U-shaped types in surface area.

Cortical thickness and surface area analyses showed different type distributions in our study. Previous studies have reported on the differences between cortical thickness and surface area analysis. Panizzon et al. reported that cortical thickness and surface area were genetically independent [28]. Storsve et al. showed a mix of positive, negative, and null relationships between changes of cortical thickness and surface area in normal aging [29]. In addition, Hogstrom et al. reported a weak negative correlation between cortical thickness and surface area [8]. Brain atrophy causing cortical thinning may not always result in a reduction of surface area. Changes of curvatures, such as increased inward bending of the surface, can occur during the process of brain atrophy [30, 31]. This may cause an increase in gyral complexity but not a decrease in surface area, despite cortical thinning—Rettmann et al. referred to this phenomenon as “cortical stretching” [30, 31]. This “cortical stretching” may help explaining the differences we found between the analysis of cortical thickness and surface area.

The maintenance of myelin is essential for normal brain function [32]. Many previous studies reported myelin breakdown associated with AD pathology [33–36]. Myelin damage or loss can contribute to the process in which neuritic plaques (NPs) and neurofibrillary tangles (NFTs) affect the manifestation of AD [32]. Bartzokis suggested that amyloid beta/tau-related homeostatic responses to age-related myelin breakdown may be one of the mechanisms leading to AD [37]. Cortico–cortical axons that myelinate late in life are the neurons most susceptible to neurodegeneration in AD [34, 38, 39]. This intracortical myelin degradation may cause the reduction of gray matter/white matter tissue contrast seen in patients with AD-related cognitive decline [40, 41]. Reduced gray matter/white matter tissue contrast may result in a shift of the gray/white matter border, which may have opposite effects on estimation of cortical thickness and surface area–changes in surface area may partly counter thickness reduction [29], and this phenomenon may also account for our results. Overall, both cortical thickness and surface area showed a tendency to decrease with cognitive decline in our study. Characteristically, surface area analysis showed inverted U-shaped types in the right fusiform, right inferior temporal, left medial orbitofrontal, left postcentral, left precentral, right supramarginal, and insula regions, unlike cortical thickness analysis. An inverted U-shaped graph indicates that structural changes in the early stages of cognitive decline are less sensitive to decrease in cognitive scores. Therefore, issues such as “cortical stretching” and shifting of gray/white matter border that may offset the descending trend of the surface area may influence brain structural measures especially in the early stages of cognitive decline. Consistent with our results, previous literature also reported that sulcal widening and gyral atrophy–that cause the reduced surface area–secondary to cortical thinning are observed throughout the brain in the late stages of cognitive decline [42].

The significant ROIs of cortical thickness analyses were distributed in the temporal and limbic lobes, and most of these ROIs were of the linear type. NFTs target temporal structures, and their distribution better corresponds to clinical impairment than the pattern and number of NPs [43, 44]. Accordingly, Braak and Braak defined six stages of NFT pathology, in which AD progresses from transentorhinal (Stage I) and entorhinal (Stage II) regions in early disease to limbic areas (Stage III, IV), then to the neocortical sensory association and prefrontal regions (Stage V), and finally to the primary sensory and motor fields (Stage VI) [45]. In addition, the temporal lobe and the parahippocampal regions are known as the late-myelinating cortical regions, which are vulnerable to the pathognomonic lesions of AD [34, 38, 39, 46]. Previous studies of structural brain MRI have reported the cortical thinning of temporal and limbic structures in AD-related cognitive decline [47–49]. Our results also showed that the cortical thickness of the temporal and limbic structures was reduced from the early to the late stage of AD-related cognitive decline in a relatively consistent manner. Therefore, according to our study, cortical thickness analysis may be preferable in assessing brain structural MRI changes of individuals with AD-related cognitive decline.

The significant ROIs of the surface area analysis were distributed in more diffuse areas in the brain as well as in the temporal and limbic lobes. Surface area analysis can be influenced by head and brain size [50]. That is, the surface area is larger in individuals with larger heads and those with larger brains, unlike cortical thickness, which is not correlated with head size measures in most regions [50]. Im et al. also reported that individual variation in brain size is mainly associated with variation in surface area, rather than cortical thickness [51]. Therefore, individual variation of head and brain size may make the surface area analysis less clear and less specific than that of cortical thickness.

The significant ROIs of volume analysis were also distributed over diffuse areas of the brain including frontal, temporal, limbic, parietal, and occipital structures. Volume might be interpreted as the product of cortical thickness and surface area. Therefore, change in both surface area as well as change in cortical thickness can drive volume change. Some previous studies even argued that change in neurons preferentially results in change in surface area rather than cortical thickness in the normal brain, because the radial axis would be mechanically stiffer than the tangential axes [52–54]. Although our study differed from those in that it targeted AD-related neurodegeneration, we believe that the effect on surface area might explain the differences between cortical thickness and volume analysis in our study. Both cortical thickness and volume analysis mainly showed linear types of descending graphs, but significant ROIs of the cortical thickness analysis were more specific to regions that are known to reflect AD-related neurodegeneration than those of volume analysis. Ashburner reported that the analysis of cortical thickness should reveal more significant results than that of volume in diseased subjects, since the disease is believed to cause cortical thinning [55]. This may be expected, considering the influence of additional variables such as changes in surface area. Accordingly, Dickerson et al. compared cortical thickness, volume, and surface area of the medial temporal lobe, and found that cortical thickness analysis better reflected episodic memory performance in patients with AD than the other two analysis metrics [50]. Our results are consistent with those reported in the previous literature.

The results of the LGI analysis were relatively less favorable than those of the other metrics. Some previous studies have compared these measures of brain structural MRI, though most of them targeted healthy normal adults [7, 8, 29, 56]. Overall, cortical thickness, volume, surface area, and LGI showed similar age-related declining patterns [7, 8, 29]. However, weak negative correlations between cortical thickness and LGI were found in some regions of the cortex [8, 56]. Rettmann et al. explained that “cortical stretching” (decreased cortical thickness with increased surface area, as mentioned above) may be the cause of the discrepancy between the analysis of cortical thickness and LGI [31]. This hypothesis may account for our results on LGI analysis. That is, cortical stretching might influence and offset the declining course of LGI and might cause relatively less significant results in LGI analysis. Our findings suggest that LGI analysis may have limited capability to reflect decreasing cognition in the progression of AD-related cognitive decline.

This study included MMSE and CERAD total scores as cognitive measures. Overall, the two analyses showed comparable distributions of significant ROIs. However, the types of descending graphs found using MMSE scores had a tendency to show a relatively elevated gradient of structural measures as a function of cognitive decline in the early stage of cognitive dysfunction than those analyzed using CERAD total scores (linear types → U-shaped types, inverted U-shaped types → linear types). These results might be attributed to the fact that MMSE scores are less sensitive than CERAD total scores to early changes in cortical structures. The CERAD total score is based on more questions, and results in a wider range of scores (0–100) than MMSE (0–30) [24, 25]. Moreover, the mean scores of CERAD total and MMSE in our NC group were 61.16 points out of 100 and 24.42 points out of 30, respectively. Therefore, MMSE scores might be less sensitive to cognitive changes than CERAD total scores, especially in the early stage of AD-related cognitive decline, due to a ceiling effect. MMSE is a simple screening tool for evaluating cognitive dysfunction [25], whereas CERAD is a comprehensive battery of neurocognitive tests [22]. Considering this difference, our results may be natural. Our findings suggest that it may be necessary to consider tools for cognitive assessment when analyzing the correlations between changes in brain structural MRI and cognitive dysfunction.

This study has several strengths and clinical implications, since it extends our knowledge of the metrics of analysis of brain structural MRI by comparing the analysis of cortical thickness, volume, surface area, and LGI. In particular, we adopted cognitive decline as a variable for analyzing the decrease in brain structural measures, unlike other studies using age, by recruiting individuals with NC, MCI, and AD [8, 29, 56]. This may help clinicians assess brain structural MRI of individuals with AD-related cognitive decline. Moreover, relatively few studies have considered LGI analysis in patients with MCI and AD compared to other metrics. This study may contribute to our understanding of use of LGI analysis for assessing brain structural MRI of individuals with AD-related cognitive decline, even if the LGI analysis did not yield favorable results. Furthermore, we identified the differences between the use of MMSE and CERAD total scores in analyzing correlations between changes of brain structural MRI and cognitive dysfunction. This may also be a noteworthy finding of our study.

There are also several limitations to this study. First, most of the participants were females, although the male-to-female ratio was not significantly different between the NC, MCI, and AD groups. Second, our study only used clinical diagnosis: The neuropathology of the subjects was not confirmed by biomarkers such as amyloid-positron emission tomography and cerebrospinal fluid. Third, this study had a cross-sectional design, thus NC, MCI, and AD groups consisted of different participants. Further longitudinal studies including brain structural MRI at various time-points during the progression of cognitive decline will help to validate our results. In addition, there are a few technical limitations. First, the automated preprocessing pipeline of FreeSurfer can over- or under-estimate the cortical measurements. When its skull strip step includes non-brain tissues, the cortical thickness can be over-estimated. Also, it has been reported that both the operating system and the FreeSurfer version can affect the results [57]. Thus, we used computers with the same operating systems, chipsets, and FreeSurfer version. Also, we visually validated the intermediate FreeSurfer results to prevent invalid pre-processing. Second, the under-estimation of cortical gray matter volume by FreeSurfer is a known issue [19]. Although this problem was resolved in the recent versions of FreeSurfer, we consistently used the same but old version (v5.3.0) to ensure the integrity of data. Third, we employed the ROI-based analysis instead of the vertex-wise analysis which enables cortical mapping of the analysis results. However, our main aim is to distinguishing the degeneration patterns, we tried to keep simple. Also, the ROI-based analysis may reduce the effects of the artifacts which lead inaccurate estimation of cortical measurements.

## Conclusion

Our study compared four brain structural MRI metrics of analysis, using MMSE/CERAD total scores and 68 ROIs in the different stages of AD-related cognitive decline. Although the morphometric measures—cortical thickness, volume, surface area, and LGI—are linked and inter-related to each other by mathematical equations, the types of descending graphs and significant ROIs were different for the various measures considered in our study. The results of this study indicate that cortical thickness analysis may be preferable in assessing brain structural MRI changes of individuals with AD-related cognitive decline, whereas LGI analysis may have limited capability to reflect cognitive decrease. Moreover, CERAD total scores were more sensitive to early changes of cortical structures than MMSE scores. Our findings may provide a reference for future studies on brain structural analysis in neurodegenerative diseases, as well as help to determine the optimal brain structural MRI analysis metrics for older adults in the course of AD-related cognitive decline.

## Supporting information

S1 Fig Procedure for determining a type of a certain region. Procedure for determining a type of a certain region. We first fit a general linear model (GLM) with the term of score^2^ (i.e. MMSE or CERAD total) to the cortical measurement of a certain region (i.e. cortical thickness, volume, surface area and local gyrification index). When its goodness-of-fit is good enough (i.e. R^2^> = 0.26) and its coefficient of the second-order term β_1_ is significant, the type of the region is determined as a U-shaped type or an inverted U-shaped depending on the sign of the coefficient β_1_. Otherwise, we fit a simpler GLM without the score^2^ term. When its goodness-of-fit is good enough and its coefficient α_1_ is significant, the type of the region is determined as a linear type.
(TIF)

S1 Table GLM analysis of cortical thickness measurement and CERAD total scores. ^a^ coefficient β_1_ that is for the score^2^; ^b^ p-value from the F-test for the coefficient β_1_; ^c^ coefficient α_1_ that is for the score of the model w/o score^2^; ^d^ p-value from the coefficient α_1_; Bold represents significant results.
(PDF)

S2 Table GLM analysis of cortical volume measurement and CERAD total scores. ^a^ coefficient β_1_ that is for the score^2^; ^b^ p-value from the F-test for the coefficient β_1_; ^c^ coefficient α_1_ that is for the score of the model w/o score^2^; ^d^ p-value from the coefficient α_1_; Bold represents significant results.
(PDF)

S3 Table GLM analysis of surface area measurement and CERAD total scores. ^a^ coefficient β_1_ that is for the score^2^; ^b^ p-value from the F-test for the coefficient β_1_; ^c^ coefficient α_1_ that is for the score of the model w/o score^2^; ^d^ p-value from the coefficient α_1_; Bold represents significant results.
(PDF)

S4 Table GLM analysis of LGI measurement and CERAD total scores. ^a^ coefficient β_1_ that is for the score^2^; ^b^ p-value from the F-test for the coefficient β_1_; ^c^ coefficient α_1_ that is for the score of the model w/o score^2^; ^d^ p-value from the coefficient α_1_; Bold represents significant results.
(PDF)

S5 Table GLM analysis of cortical thickness measurement and MMSE scores. ^a^ coefficient β_1_ that is for the score^2^; ^b^ p-value from the F-test for the coefficient β_1_; ^c^ coefficient α_1_ that is for the score of the model w/o score^2^; ^d^ p-value from the coefficient α_1_; Bold represents significant results.
(PDF)

S6 Table GLM analysis of cortical volume measurement and MMSE scores. ^a^ coefficient β_1_ that is for the score^2^; ^b^ p-value from the F-test for the coefficient β_1_; ^c^ coefficient α_1_ that is for the score of the model w/o score^2^; ^d^ p-value from the coefficient α_1_; Bold represents significant results.
(PDF)

S7 Table GLM analysis of surface area measurement and MMSE scores. ^a^ coefficient β_1_ that is for the score^2^; ^b^ p-value from the F-test for the coefficient β_1_; ^c^ coefficient α_1_ that is for the score of the model w/o score^2^; ^d^ p-value from the coefficient α_1_; Bold represents significant results.
(PDF)

S8 Table GLM analysis of LGI measurement and MMSE scores. ^a^ coefficient β_1_ that is for the score^2^; ^b^ p-value from the F-test for the coefficient β_1_; ^c^ coefficient α_1_ that is for the score of the model w/o score^2^; ^d^ p-value from the coefficient α_1_; Bold represents significant results.
(PDF)
