# Correction: Comparison of neurodegenerative types using different brain MRI analysis metrics in older adults with normal cognition, mild cognitive impairment, and Alzheimer’s dementia

**DOI:** 10.1371/journal.pone.0222852

**Published:** 2019-09-17

**Authors:** The PLOS ONE Staff

There are errors in the Funding statement. The publisher apologies for these errors. The correct Funding statement is as follows: This work was supported by the Korea Health Technology R&D Project through the Korea Health Industry Development Institute (KHIDI) that was funded by the Ministry of Health & Welfare, Republic of Korea (HG Jeong, HC15C1509, HG Jeong & CE Han, HI19C0645); the Basic Science Research Program through the National Research Foundation of Korea (NRF), funded by the Ministry of Science and ICT (HG Jeong, NRF-2015R1C1A1A01052172); the Basic Science Research Program through the National Research Foundation of Korea (NRF) funded by the Ministry of Education of the Government of the Republic of Korea (CE Han, 2016R1D1A1B03934990). The URLs are https://www.khidi.or.kr/kps and http://nrf.re.kr/index. The funders had no role in study design, data collection and analysis, decision to publish, or preparation of the manuscript.
