# Supplementary figures and images for: Comparison of neurodegenerative types using different brain MRI analysis metrics in older adults with normal cognition, mild cognitive impairment, and Alzheimer’s dementia
Source: PLoS One. 2019 Aug 1;14(8):e0220739. doi: 10.1371/journal.pone.0220739 (PMC6675320; doi:10.1371/journal.pone.0220739)

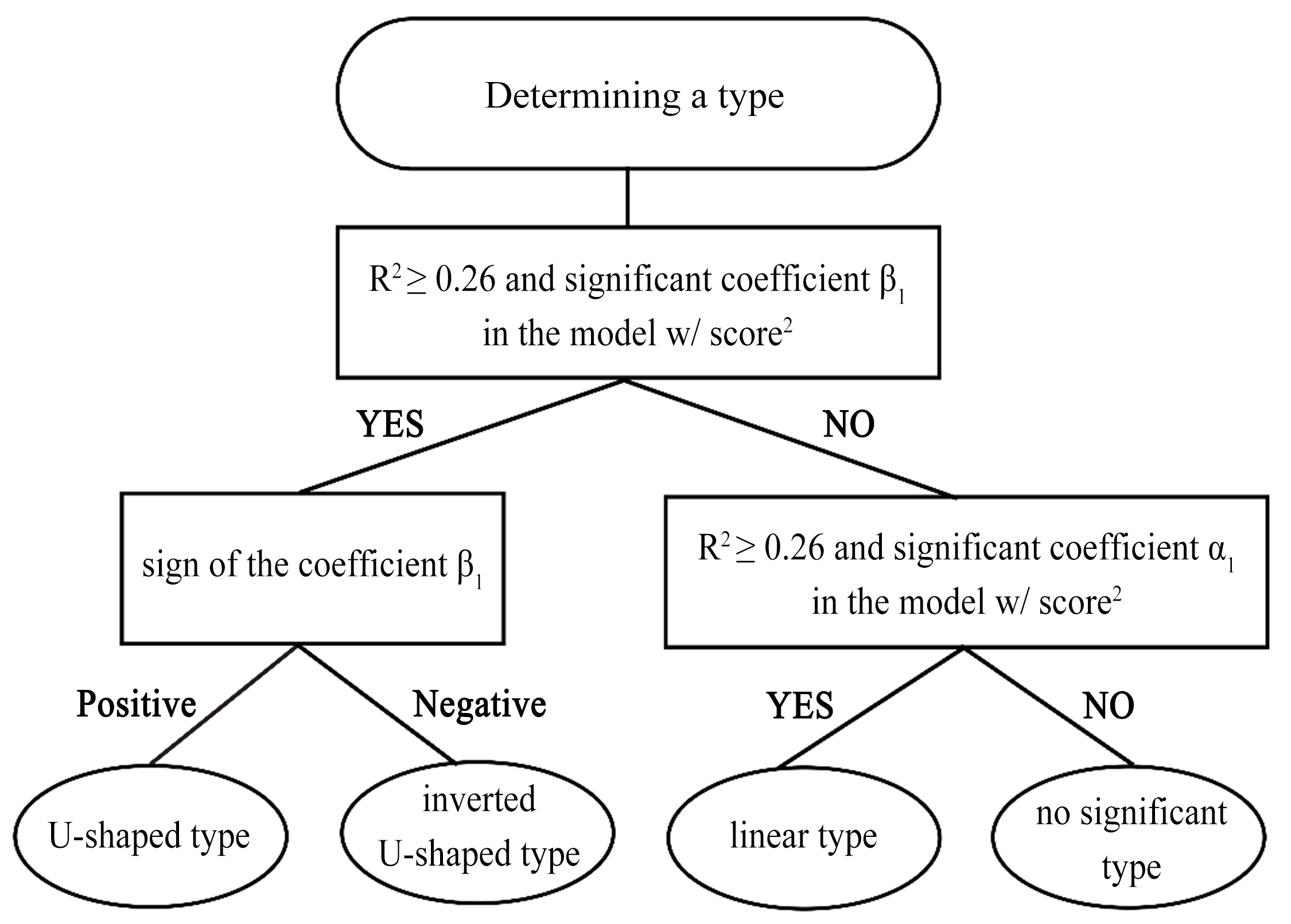

Supplement: S1 Fig — Procedure for determining a type of a certain region. We first fit a general linear model (GLM) with the term of score2 (i.e. MMSE or CERAD total) to the cortical measurement of a certain region (i.e. cortical thickness, volume, surface area and local gyrification index). When its goodness-of-fit is good enough (i.e. R2> = 0.26) and its coefficient of the second-order term β1 is significant, the type of the region is determined as a U-shaped type or an inverted U-shaped depending on the sign of the coefficient β1. Otherwise, we fit a simpler GLM without the score2 term. When its goodness-of-fit is good enough and its coefficient α1 is significant, the type of the region is determined as a linear type. (TIF) [file pone.0220739.s001.tif]
