# Supplementary material for: Comparison of neurodegenerative types using different brain MRI analysis metrics in older adults with normal cognition, mild cognitive impairment, and Alzheimer’s dementia
Source: PLoS One. 2019 Aug 1;14(8):e0220739. doi: 10.1371/journal.pone.0220739 (PMC6675320; doi:10.1371/journal.pone.0220739)
Supplement: S1 Table — a coefficient β1 that is for the score2; b p-value from the F-test for the coefficient β1; c coefficient α1 that is for the score of the model w/o score2; d p-value from the coefficient α1; Bold represents significant results. (PDF) [file pone.0220739.s002.pdf]

	Measure	Type	Model w/ score^2			Model w/o score^2			Measure	Type	Model w/ score^2			Model w/o score^2		
			β_1[a]	p-	R^2	α_1[c]	p-	R^2			β_1[a]	p-	R^2	α_1[c]	p-	R^2
bankssts	T_lh	N/A	0.0001	0.154	0.16	0.0042	**0.004**	0.14	T_rh	N/A	0.0000	0.688	0.16	0.0050	**0.001**	0.16
caudalanteriorcingulate	T_lh	N/A	0.0001	0.221	0.07	-0.0001	0.969	0.05	T_rh	N/A	0.0000	0.576	0.10	-	0.943	0.09
caudalmiddlefrontal	T_lh	N/A	0.0001	**0.015**	0.11	0.0013	0.278	0.05	T_rh	N/A	0.0001	0.139	0.11	0.0027	**0.031**	0.09
cuneus	T_lh	N/A	0.0000	0.501	0.11	0.0006	0.480	0.10	T_rh	N/A	0.0001	0.104	0.10	0.0002	0.810	0.08
entorhinal	T_lh	**Linear**	-0.0001	0.550	**0.43**	0.0193	**0.000**	**0.42**	T_rh	**Linear**	0.0000	0.767	**0.40**	0.0207	**0.000**	**0.40**
fusiform	T_lh	**Linear**	0.0001	0.169	**0.31**	0.0068	**0.000**	**0.29**	T_rh	N/A	0.0001	0.234	0.22	0.0054	**0.000**	0.21
inferiorparietal	T_lh	N/A	0.0002	**0.014**	0.17	0.0031	**0.029**	0.11	T_rh	N/A	0.0001	**0.044**	0.18	0.0041	**0.002**	0.14
inferiortemporal	T_lh	N/A	0.0001	0.091	0.26	0.0065	**0.000**	0.23	T_rh	N/A	0.0001	0.350	0.26	0.0073	**0.000**	0.25
isthmuscingulate	T_lh	**U**	0.0002	**0.012**	**0.31**	-	-	-	T_rh	N/A	0.0001	0.123	0.25	0.0054	**0.000**	0.23
lateraloccipital	T_lh	N/A	0.0001	0.152	0.21	0.0018	0.094	0.19	T_rh	N/A	0.0001	0.196	0.20	0.0022	**0.044**	0.18
lateralorbitofrontal	T_lh	N/A	0.0000	0.420	0.10	0.0032	**0.017**	0.09	T_rh	N/A	0.0000	0.512	0.17	0.0046	**0.000**	0.17
lingual	T_lh	N/A	0.0000	0.657	0.18	0.0018	**0.014**	0.18	T_rh	N/A	0.0000	0.254	0.15	0.0010	0.206	0.14
medialorbitofrontal	T_lh	N/A	0.0001	0.082	0.15	0.0027	**0.044**	0.12	T_rh	N/A	0.0001	0.303	0.10	0.0036	**0.019**	0.09
middletemporal	T_lh	N/A	0.0000	0.590	0.24	0.0069	**0.000**	0.24	T_rh	**Linear**	0.0000	0.609	**0.27**	0.0076	**0.000**	**0.26**
parahippocampal	T_lh	**Linear**	-0.0001	0.423	**0.30**	0.0074	**0.000**	**0.30**	T_rh	N/A	-	0.325	0.26	0.0064	**0.000**	0.25
paracentral	T_lh	N/A	0.0001	0.167	0.14	0.0009	0.564	0.12	T_rh	N/A	0.0001	0.179	0.15	0.0009	0.513	0.13
parsopercularis	T_lh	N/A	0.0000	0.718	0.10	0.0015	0.136	0.10	T_rh	N/A	0.0001	**0.040**	0.16	0.0017	0.115	0.12
parsorbitalis	T_lh	N/A	0.0000	0.601	0.10	0.0028	0.070	0.10	T_rh	N/A	0.0001	0.063	0.11	0.0013	0.361	0.08
parstriangularis	T_lh	N/A	0.0000	0.373	0.08	0.0014	0.190	0.07	T_rh	N/A	0.0001	0.057	0.12	0.0025	**0.045**	0.08
pericalcarine	T_lh	N/A	0.0000	0.616	0.07	0.0008	0.237	0.07	T_rh	N/A	0.0000	0.989	0.07	0.0013	0.066	0.07
postcentral	T_lh	N/A	0.0001	0.109	0.12	0.0012	0.239	0.10	T_rh	N/A	0.0001	0.222	0.15	0.0023	**0.019**	0.14
posteriorcingulate	T_lh	N/A	0.0002	**0.010**	0.14	0.0025	0.072	0.08	T_rh	N/A	0.0001	0.255	0.17	0.0037	**0.004**	0.16
precentral	T_lh	N/A	0.0001	**0.013**	0.21	0.0009	0.511	0.16	T_rh	N/A	0.0001	0.076	0.18	0.0017	0.212	0.15
precuneus	T_lh	N/A	0.0001	**0.044**	0.15	0.0034	**0.011**	0.11	T_rh	N/A	0.0001	0.091	0.17	0.0036	**0.004**	0.14
rostralanteriorcingulate	T_lh	N/A	0.0000	0.586	0.18	0.0054	**0.001**	0.18	T_rh	N/A	0.0000	0.650	0.09	0.0030	0.084	0.09
rostralmiddlefrontal	T_lh	N/A	0.0001	**0.040**	0.19	0.0022	**0.038**	0.15	T_rh	N/A	0.0001	**0.017**	0.15	0.0015	0.147	0.10
superiorfrontal	T_lh	N/A	0.0001	**0.038**	0.18	0.0024	0.068	0.14	T_rh	N/A	0.0001	**0.037**	0.16	0.0026	**0.040**	0.12
superiorparietal	T_lh	N/A	0.0001	**0.044**	0.09	0.0007	0.568	0.04	T_rh	N/A	0.0001	0.092	0.08	0.0016	0.188	0.05
superiortemporal	T_lh	**Linear**	0.0000	0.996	**0.34**	0.0056	**0.000**	**0.34**	T_rh	**Linear**	0.0000	0.922	**0.31**	0.0072	**0.000**	**0.31**
supramarginal	T_lh	N/A	0.0001	**0.040**	0.24	0.0039	**0.001**	0.20	T_rh	N/A	0.0001	0.086	0.21	0.0045	**0.001**	0.19
frontalpole	T_lh	N/A	0.0002	**0.039**	0.13	0.0037	0.075	0.09	T_rh	N/A	0.0000	0.855	0.10	0.0032	0.072	0.10
temporalpole	T_lh	N/A	0.0000	0.942	0.20	0.0111	**0.000**	0.20	T_rh	N/A	0.0002	0.134	0.24	0.0130	**0.000**	0.22
transversetemporal	T_lh	N/A	0.0000	0.772	0.17	0.0025	0.102	0.17	T_rh	N/A	0.0001	0.326	0.19	0.0045	**0.008**	0.18
insula	T_lh	N/A	0.0000	0.730	0.20	0.0038	**0.002**	0.20	T_rh	**Linear**	0.0001	0.360	**0.29**	0.0055	**0.000**	**0.28**
