# Supplementary material for: Comparison of neurodegenerative types using different brain MRI analysis metrics in older adults with normal cognition, mild cognitive impairment, and Alzheimer’s dementia
Source: PLoS One. 2019 Aug 1;14(8):e0220739. doi: 10.1371/journal.pone.0220739 (PMC6675320; doi:10.1371/journal.pone.0220739)
Supplement: S2 Table — a coefficient β1 that is for the score2; b p-value from the F-test for the coefficient β1; c coefficient α1 that is for the score of the model w/o score2; d p-value from the coefficient α1; Bold represents significant results. (PDF) [file pone.0220739.s003.pdf]

			Model w/ score^2			Model w/o score^2					Model w/ score^2			Model w/o score^2		
	Measure	Type	β_1[a]	p-	R^2	α_1[c]	p-	R^2	Measure	Type	β_1[a]	p-	R^2	α_1[c]	p-	R^2
bankssts	V_lh	**Linear**	0.0133	0.891	**0.32**	10.2986	**0.000**	**0.32**	V_rh	**Linear**	-	0.210	**0.31**	5.5255	**0.014**	**0.29**
caudalanteriorcingulate	V_lh	N/A	-0.0240	0.815	0.04	2.5348	0.281	0.04	V_rh	N/A	-	0.084	0.09	4.2180	0.154	0.06
caudalmiddlefrontal	V_lh	N/A	0.5422	**0.011**	0.22	1.1483	0.816	0.16	V_rh	N/A	0.1001	0.608	0.26	13.2011	**0.004**	0.26
cuneus	V_lh	N/A	0.0353	0.697	0.19	4.7905	**0.022**	0.19	V_rh	N/A	0.0558	0.550	0.21	2.7590	0.198	0.21
entorhinal	V_lh	**Linear**	-0.1550	0.111	**0.31**	9.7672	**0.000**	**0.29**	V_rh	N/A	-	0.252	0.25	8.9132	**0.000**	0.24
fusiform	V_lh	**Linear**	0.0423	0.898	**0.36**	32.8547	**0.000**	**0.36**	V_rh	**Linear**	-	0.535	**0.29**	23.7488	**0.001**	**0.28**
inferiorparietal	V_lh	**Linear**	0.5873	0.135	**0.35**	40.0467	**0.000**	**0.33**	V_rh	**U**	1.0503	**0.021**	**0.43**	-	-	-
inferiortemporal	V_lh	**Linear**	0.0870	0.786	**0.41**	35.4936	**0.000**	**0.41**	V_rh	**Linear**	-	0.774	**0.45**	50.9621	**0.000**	**0.45**
isthmuscingulate	V_lh	N/A	0.0420	0.634	0.25	9.2648	**0.000**	0.25	V_rh	**Linear**	0.0338	0.683	**0.30**	8.6199	**0.000**	**0.30**
lateraloccipital	V_lh	N/A	0.5632	0.110	0.22	8.4610	0.298	0.19	V_rh	N/A	0.1443	0.678	**0.30**	14.2128	0.076	**0.30**
lateralorbitofrontal	V_lh	**Linear**	-0.0691	0.676	**0.33**	9.4125	**0.014**	**0.32**	V_rh	N/A	-	0.953	0.25	9.1863	**0.019**	0.25
lingual	V_lh	**Linear**	-0.1452	0.455	**0.27**	16.0013	**0.000**	**0.27**	V_rh	N/A	-	0.983	0.17	5.7471	0.187	0.17
medialorbitofrontal	V_lh	N/A	-0.1252	0.409	0.25	5.6550	0.106	0.25	V_rh	N/A	-	0.754	0.23	8.0461	**0.009**	0.23
middletemporal	V_lh	**Linear**	-0.2003	0.530	**0.42**	46.3220	**0.000**	**0.42**	V_rh	**Linear**	0.0082	0.983	**0.40**	49.2776	**0.000**	**0.40**
parahippocampal	V_lh	**Linear**	-0.0425	0.558	**0.42**	8.9466	**0.000**	**0.41**	V_rh	N/A	-	0.379	0.22	6.0928	**0.001**	0.21
paracentral	V_lh	N/A	0.1086	0.359	0.24	0.5542	0.838	0.24	V_rh	N/A	0.1382	0.287	**0.31**	1.7747	0.551	**0.30**
parsopercularis	V_lh	N/A	0.0799	0.594	0.13	5.3298	0.122	0.12	V_rh	N/A	0.0387	0.790	0.17	2.3257	0.483	0.17
parsorbitalis	V_lh	N/A	-0.0183	0.794	0.06	3.0922	0.056	0.06	V_rh	N/A	0.0981	0.267	0.13	3.8761	0.058	0.12
parstriangularis	V_lh	N/A	-0.0147	0.906	0.04	4.4091	0.121	0.04	V_rh	N/A	-	0.876	0.08	5.0420	0.181	0.08
pericalcarine	V_lh	N/A	0.0887	0.264	0.11	3.2017	0.081	0.09	V_rh	N/A	-	0.393	0.11	1.3259	0.507	0.10
postcentral	V_lh	N/A	-0.2249	0.347	0.25	17.4254	**0.002**	0.25	V_rh	N/A	0.1844	0.495	0.23	19.6715	**0.002**	0.23
posteriorcingulate	V_lh	N/A	0.0760	0.497	0.21	7.4156	**0.005**	0.21	V_rh	N/A	-	0.882	0.22	9.9657	**0.000**	0.22
precentral	V_lh	N/A	0.3333	0.351	**0.31**	15.5047	0.060	**0.30**	V_rh	N/A	0.3381	0.348	0.24	8.8909	0.282	0.23
precuneus	V_lh	N/A	0.1816	0.543	0.23	21.8840	**0.002**	0.23	V_rh	**Linear**	0.2488	0.388	**0.33**	27.9509	**0.000**	**0.32**
rostralanteriorcingulate	V_lh	N/A	-0.1193	0.259	0.15	5.8914	**0.017**	0.14	V_rh	N/A	-	0.109	0.16	5.7081	**0.018**	0.14
rostralmiddlefrontal	V_lh	**Linear**	0.2536	0.510	**0.31**	26.8146	**0.003**	**0.30**	V_rh	**Linear**	0.7518	0.058	**0.39**	23.1850	**0.013**	**0.36**
superiorfrontal	V_lh	**Linear**	0.3582	0.443	**0.41**	26.1758	**0.016**	**0.40**	V_rh	**Linear**	0.0721	0.881	**0.28**	30.9061	**0.006**	**0.28**
superiorparietal	V_lh	N/A	0.3453	0.333	0.19	14.6655	0.075	0.18	V_rh	N/A	0.2329	0.536	0.17	19.7638	**0.023**	0.17
superiortemporal	V_lh	**Linear**	-0.0669	0.800	**0.45**	30.1260	**0.000**	**0.45**	V_rh	**Linear**	-	0.336	**0.36**	34.1643	**0.000**	**0.35**
supramarginal	V_lh	**Linear**	0.0438	0.885	**0.28**	25.9857	**0.000**	**0.28**	V_rh	N/A	-	0.765	0.24	27.2700	**0.000**	0.24
frontalpole	V_lh	N/A	0.0708	0.091	0.07	1.3065	0.177	0.04	V_rh	N/A	0.0093	0.850	0.07	1.5962	0.155	0.07
temporalpole	V_lh	N/A	-0.0842	0.418	0.16	8.0593	**0.001**	0.15	V_rh	N/A	0.1499	0.166	0.16	8.7661	**0.001**	0.14
transversetemporal	V_lh	N/A	0.0049	0.918	0.22	2.7349	**0.014**	0.22	V_rh	N/A	0.0188	0.595	0.17	2.0162	**0.014**	0.17
insula	V_lh	**Linear**	-0.1861	0.222	**0.37**	12.5238	**0.001**	**0.36**	V_rh	**Linear**	-	0.466	**0.30**	18.3831	**0.000**	**0.30**
