# Supplementary material for: Comparison of neurodegenerative types using different brain MRI analysis metrics in older adults with normal cognition, mild cognitive impairment, and Alzheimer’s dementia
Source: PLoS One. 2019 Aug 1;14(8):e0220739. doi: 10.1371/journal.pone.0220739 (PMC6675320; doi:10.1371/journal.pone.0220739)
Supplement: S3 Table — a coefficient β1 that is for the score2; b p-value from the F-test for the coefficient β1; c coefficient α1 that is for the score of the model w/o score2; d p-value from the coefficient α1; Bold represents significant results. (PDF) [file pone.0220739.s004.pdf]

			Model w/ score²			Model w/o score²					Model w/ score²					Mode
	Measur	Type	β_1[a]	p-	R^2	α_1[c]	p-	R^2	Measur	Type	β_1[a]	p-	R^2	α_1[c]	p-	R^2
bankssts	S_lh	**Linear**	-	0.390	**0.3**	2.6594	**0.00**	**0.3**	S_rh	N/A	-	0.167	**0.3**	0.7392	0.302	**0.30**
caudalanteriorcingulat	S_lh	N/A	-	0.263	0.14	0.8877	0.220	0.13	S_rh	N/A	-	**0.03**	0.19	1.2130	0.162	0.15
caudalmiddlefrontal	S_lh	N/A	0.0623	0.346	**0.2**	-0.4094	0.787	**0.2**	S_rh	**Linear**	-	0.309	**0.3**	3.0224	**0.03**	**0.34**
cuneus	S_lh	N/A	0.0073	0.875	0.21	2.2403	**0.03**	0.21	S_rh	N/A	-	0.656	0.20	1.3300	0.198	0.20
entorhinal	S_lh	N/A	-	**0.01**	0.16	-0.4272	0.398	0.11	S_rh	N/A	-	0.078	0.21	-0.5869	0.151	0.19
fusiform	S_lh	N/A	-	0.156	**0.3**	3.5536	0.094	**0.3**	S_rh	**inverte**	-	**0.03**	**0.3**	-	-	-
inferiorparietal	S_lh	**Linear**	-	0.285	**0.5**	10.367	**0.00**	**0.5**	S_rh	**Linear**	0.062	0.641	**0.4**	12.796	**0.00**	**0.44**
inferiortemporal	S_lh	**Linear**	-	0.121	**0.4**	4.2665	**0.04**	**0.4**	S_rh	**inverte**	-	**0.03**	**0.5**	-	-	-
isthmuscingulate	S_lh	N/A	-	0.060	0.26	1.0397	0.189	0.23	S_rh	N/A	-	0.232	**0.3**	1.3095	0.087	**0.32**
lateraloccipital	S_lh	N/A	0.0291	0.811	**0.2**	-0.5291	0.849	**0.2**	S_rh	N/A	-	0.563	**0.3**	1.6584	0.562	**0.29**
lateralorbitofrontal	S_lh	N/A	-	0.098	**0.3**	0.7963	0.574	**0.3**	S_rh	N/A	-	0.184	**0.2**	-0.3747	0.792	**0.28**
lingual	S_lh	N/A	-	0.236	0.25	6.0413	**0.00**	0.24	S_rh	N/A	-	0.388	0.16	1.5222	0.413	0.15
medialorbitofrontal	S_lh	**inverted**	-	**0.00**	**0.4**	-	-	-	S_rh	N/A	-	0.051	**0.3**	0.1037	0.919	**0.30**
middletemporal	S_lh	**Linear**	-	0.181	**0.4**	7.2448	**0.00**	**0.4**	S_rh	**Linear**	-	0.126	**0.4**	7.4657	**0.00**	**0.46**
parahippocampal	S_lh	**Linear**	-	0.592	**0.4**	1.1444	**0.00**	**0.4**	S_rh	N/A	-	0.525	0.12	0.6631	0.182	0.12
paracentral	S_lh	N/A	-	0.634	0.17	-0.4945	0.629	0.17	S_rh	N/A	-	0.743	**0.2**	-0.3764	0.725	**0.29**
parsopercularis	S_lh	N/A	0.0150	0.763	0.17	1.4946	0.189	0.17	S_rh	N/A	-	0.312	0.22	0.3675	0.752	0.21
parsorbitalis	S_lh	N/A	-	0.444	0.16	0.4617	0.275	0.16	S_rh	**Linear**	-	0.443	**0.2**	1.2752	**0.03**	**0.27**
parstriangularis	S_lh	N/A	-	0.424	0.08	1.1458	0.255	0.08	S_rh	N/A	-	0.065	0.19	0.6253	0.609	0.16
pericalcarine	S_lh	N/A	0.0374	0.464	0.09	1.5565	0.185	0.09	S_rh	N/A	-	0.528	0.11	-0.3441	0.780	0.11
postcentral	S_lh	**inverted**	-	**0.00**	**0.3**	-	-	-	S_rh	N/A	-	0.354	**0.2**	3.7524	0.133	**0.26**
posteriorcingulate	S_lh	N/A	-	0.187	**0.2**	1.2903	0.125	**0.2**	S_rh	N/A	-	0.170	**0.2**	2.1907	**0.01**	0.25
precentral	S_lh	**inverted**	-	**0.04**	**0.4**	-	-	-	S_rh	N/A	-	0.237	**0.3**	0.5515	0.817	**0.35**
precuneus	S_lh	N/A	-	0.147	0.25	3.5723	0.111	0.23	S_rh	N/A	-	0.284	**0.2**	5.4897	**0.02**	0.26
rostralanteriorcingulat	S_lh	N/A	-	0.222	0.20	1.0263	0.205	0.19	S_rh	N/A	-	**0.04**	0.20	1.0637	0.165	0.17
rostralmiddlefrontal	S_lh	**Linear**	-	0.174	**0.4**	6.8630	**0.03**	**0.4**	S_rh	**Linear**	-	0.682	**0.4**	6.8854	**0.04**	**0.47**
superiorfrontal	S_lh	N/A	-	0.093	**0.5**	3.6394	0.263	**0.5**	S_rh	N/A	-	0.050	**0.4**	4.8917	0.120	**0.45**
superiorparietal	S_lh	N/A	-	0.291	0.21	5.1463	0.092	0.20	S_rh	N/A	-	0.218	**0.2**	4.6115	0.072	**0.28**
superiortemporal	S_lh	N/A	-	0.219	**0.4**	3.2420	0.062	**0.4**	S_rh	N/A	-	0.063	**0.3**	2.8669	0.115	**0.33**
supramarginal	S_lh	N/A	-	0.070	**0.3**	4.4078	0.061	**0.3**	S_rh	**inverte**	-	**0.03**	**0.2**	-	-	-
frontalpole	S_lh	N/A	-	0.678	0.03	0.2097	0.265	0.02	S_rh	N/A	0.001	0.870	0.12	0.0854	0.709	0.12
temporalpole	S_lh	N/A	-	**0.01**	0.19	0.6594	**0.04**	0.13	S_rh	N/A	-	0.395	0.15	0.2008	0.589	0.15
transversetemporal	S_lh	N/A	0.0031	0.843	0.19	0.4221	0.242	0.19	S_rh	N/A	-	0.757	0.12	0.0514	0.850	0.12
insula	S_lh	**inverted**	-	**0.02**	**0.4**	-	-	-	S_rh	**inverte**	-	**0.00**	**0.3**	-	-	-
