# Supplementary material for: Comparison of neurodegenerative types using different brain MRI analysis metrics in older adults with normal cognition, mild cognitive impairment, and Alzheimer’s dementia
Source: PLoS One. 2019 Aug 1;14(8):e0220739. doi: 10.1371/journal.pone.0220739 (PMC6675320; doi:10.1371/journal.pone.0220739)
Supplement: S4 Table — a coefficient β1 that is for the score2; b p-value from the F-test for the coefficient β1; c coefficient α1 that is for the score of the model w/o score2; d p-value from the coefficient α1; Bold represents significant results. (PDF) [file pone.0220739.s005.pdf]

	Measure	Type	Model w/ score^2			Model w/o score^2			Measure	Type	Model w/ score^2			Model w/o score^2		
			β_1[a]	p-	R^2	α_1[c]	p-	R^2			β_1[a]	p-	R^2	α_1[c]	p-	R^2
bankssts	LGI_lh	N/A	-0.0001	**0.027**	0.25	0.0050	**0.000**	0.21	LGI_rh	N/A	-	0.061	0.19	0.0040	**0.000**	0.16
caudalanteriorcingulate	LGI_lh	N/A	0.0000	0.887	0.05	-0.0002	0.719	0.05	LGI_rh	N/A	0.0000	0.525	0.14	0.0001	0.864	0.14
caudalmiddlefrontal	LGI_lh	N/A	0.0000	0.461	0.06	0.0005	0.625	0.05	LGI_rh	N/A	0.0000	0.570	0.09	0.0014	0.099	0.09
cuneus	LGI_lh	N/A	0.0000	0.781	0.06	0.0017	0.119	0.06	LGI_rh	N/A	0.0000	0.577	0.02	0.0003	0.787	0.01
entorhinal	LGI_lh	N/A	0.0000	0.383	0.11	-0.0003	0.657	0.10	LGI_rh	N/A	0.0000	0.714	0.10	0.0009	0.247	0.09
fusiform	LGI_lh	N/A	0.0000	0.866	0.14	0.0010	0.095	0.14	LGI_rh	N/A	0.0000	0.332	0.17	0.0011	**0.044**	0.16
inferiorparietal	LGI_lh	N/A	0.0000	0.492	0.09	0.0015	**0.033**	0.09	LGI_rh	N/A	0.0000	0.777	0.15	0.0018	**0.021**	0.15
inferiortemporal	LGI_lh	N/A	0.0000	0.148	0.05	0.0010	0.118	0.03	LGI_rh	N/A	0.0000	0.679	0.05	0.0005	0.405	0.05
isthmuscingulate	LGI_lh	N/A	0.0000	0.719	0.13	0.0015	0.137	0.13	LGI_rh	N/A	0.0000	0.656	0.10	0.0014	0.227	0.10
lateraloccipital	LGI_lh	N/A	0.0000	0.671	0.07	0.0009	0.163	0.07	LGI_rh	N/A	0.0000	0.973	0.05	0.0010	0.124	0.05
lateralorbitofrontal	LGI_lh	N/A	0.0000	0.952	0.12	0.0010	0.141	0.12	LGI_rh	N/A	0.0000	0.147	0.23	0.0010	0.101	0.22
lingual	LGI_lh	N/A	0.0000	0.943	0.07	0.0011	0.165	0.07	LGI_rh	N/A	0.0000	0.367	0.09	0.0008	0.333	0.08
medialorbitofrontal	LGI_lh	N/A	0.0000	0.302	0.05	-0.0003	0.512	0.04	LGI_rh	N/A	0.0000	**0.035**	0.10	-	0.847	0.05
middletemporal	LGI_lh	N/A	-0.0001	**0.021**	0.14	0.0032	**0.006**	0.09	LGI_rh	N/A	-	0.210	0.10	0.0024	**0.014**	0.09
parahippocampal	LGI_lh	N/A	0.0000	0.514	0.22	0.0008	0.269	0.22	LGI_rh	N/A	0.0000	0.158	0.19	0.0009	0.258	0.17
paracentral	LGI_lh	N/A	0.0000	0.541	0.02	0.0007	0.373	0.02	LGI_rh	N/A	0.0000	0.543	0.02	0.0004	0.624	0.02
parsopercularis	LGI_lh	N/A	-0.0001	0.293	0.13	0.0036	**0.023**	0.12	LGI_rh	N/A	-	0.172	0.15	0.0042	**0.006**	0.13
parsorbitalis	LGI_lh	N/A	-0.0001	0.266	0.16	0.0023	0.057	0.15	LGI_rh	N/A	0.0000	0.435	0.13	0.0014	0.199	0.13
parstriangularis	LGI_lh	N/A	0.0000	0.486	0.14	0.0031	**0.023**	0.14	LGI_rh	N/A	0.0000	0.909	0.08	0.0027	0.083	0.08
pericalcarine	LGI_lh	N/A	0.0000	0.469	0.05	0.0011	0.263	0.05	LGI_rh	N/A	0.0000	0.589	0.04	0.0001	0.940	0.04
postcentral	LGI_lh	N/A	-0.0001	0.181	0.16	0.0017	0.055	0.14	LGI_rh	N/A	0.0000	0.366	0.22	0.0024	**0.005**	0.21
posteriorcingulate	LGI_lh	N/A	0.0000	0.832	0.05	0.0011	0.214	0.05	LGI_rh	N/A	0.0000	0.721	0.07	0.0010	0.187	0.07
precentral	LGI_lh	N/A	0.0000	0.390	0.13	0.0018	**0.046**	0.13	LGI_rh	N/A	0.0000	0.635	0.18	0.0024	**0.003**	0.18
precuneus	LGI_lh	N/A	0.0000	0.819	0.09	0.0017	0.094	0.09	LGI_rh	N/A	0.0000	0.712	0.04	0.0006	0.550	0.04
rostralanteriorcingulate	LGI_lh	N/A	0.0000	0.175	0.05	-0.0004	0.396	0.03	LGI_rh	N/A	0.0000	0.143	0.09	0.0000	0.966	0.07
rostralmiddlefrontal	LGI_lh	N/A	0.0000	0.277	0.10	0.0008	0.279	0.09	LGI_rh	N/A	0.0000	0.927	0.13	0.0008	0.252	0.13
superiorfrontal	LGI_lh	N/A	0.0000	0.687	0.01	-0.0003	0.558	0.01	LGI_rh	N/A	0.0000	0.654	0.06	-	0.866	0.06
superiorparietal	LGI_lh	N/A	0.0000	0.524	0.13	0.0015	**0.018**	0.12	LGI_rh	N/A	0.0000	0.907	0.04	0.0011	0.130	0.04
superiortemporal	LGI_lh	N/A	-0.0001	**0.015**	0.21	0.0044	**0.002**	0.15	LGI_rh	N/A	-	**0.044**	0.25	0.0044	**0.000**	0.21
supramarginal	LGI_lh	N/A	-0.0001	**0.021**	0.14	0.0019	0.078	0.08	LGI_rh	N/A	-	0.216	0.18	0.0026	**0.007**	0.16
frontalpole	LGI_lh	N/A	0.0000	0.285	0.07	-0.0003	0.553	0.05	LGI_rh	N/A	0.0000	0.442	0.05	-	0.756	0.04
temporalpole	LGI_lh	N/A	0.0000	0.443	0.04	-0.0002	0.759	0.03	LGI_rh	N/A	0.0000	0.743	0.07	0.0001	0.805	0.07
transversetemporal	LGI_lh	N/A	-0.0002	**0.035**	0.19	0.0044	**0.011**	0.15	LGI_rh	**inverted**	-	**0.035**	**0.26**	-	-	-
insula	LGI_lh	N/A	-0.0001	0.185	0.20	0.0042	**0.005**	0.19	LGI_rh	N/A	-	0.318	0.16	0.0042	**0.006**	0.15
