# Supplementary material for: Comparison of neurodegenerative types using different brain MRI analysis metrics in older adults with normal cognition, mild cognitive impairment, and Alzheimer’s dementia
Source: PLoS One. 2019 Aug 1;14(8):e0220739. doi: 10.1371/journal.pone.0220739 (PMC6675320; doi:10.1371/journal.pone.0220739)
Supplement: S5 Table — a coefficient β1 that is for the score2; b p-value from the F-test for the coefficient β1; c coefficient α1 that is for the score of the model w/o score2; d p-value from the coefficient α1; Bold represents significant results. (PDF) [file pone.0220739.s006.pdf]

			Model w/ score^2			Model w/o score^2					Model w/ score^2			Model w/o score^2		
	Measure	Type	β_1[a]	p-	R^2	α_1[c]	p-	R^2	Measure	Type	β_1[a]	p-	R^2	α_1[c]	p-	R^2
bankssts	T_lh	N/A	0.0003	0.459	0.18	0.0135	**0.001**	0.17	T_rh	N/A	0.0007	0.145	0.18	0.0135	**0.001**	0.16
caudalanteriorcingulate	T_lh	N/A	0.0002	0.757	0.06	0.0025	0.678	0.06	T_rh	N/A	-	0.576	0.11	0.0019	0.706	0.10
caudalmiddlefrontal	T_lh	N/A	0.0010	**0.010**	0.12	0.0027	0.412	0.06	T_rh	N/A	0.0010	**0.015**	0.13	0.0060	0.074	0.08
cuneus	T_lh	N/A	0.0004	0.144	0.14	0.0017	0.410	0.12	T_rh	N/A	0.0004	0.132	0.13	0.0015	0.554	0.11
entorhinal	T_lh	**Linear**	0.0006	0.551	**0.39**	0.0476	**0.000**	**0.39**	T_rh	**U**	0.0023	**0.049**	**0.38**	-	-	-
fusiform	T_lh	**U**	0.0011	**0.013**	**0.31**	-	-	-	T_rh	**U**	0.0012	**0.007**	**0.28**	-	-	-
inferiorparietal	T_lh	N/A	0.0010	**0.029**	0.16	0.0085	**0.026**	0.12	T_rh	N/A	0.0010	**0.017**	0.18	0.0104	**0.003**	0.13
inferiortemporal	T_lh	**U**	0.0012	**0.010**	**0.29**	-	-	-	T_rh	N/A	0.0011	**0.042**	0.25	0.0177	**0.000**	0.22
isthmuscingulate	T_lh	**U**	0.0018	**0.000**	**0.34**	-	-	-	T_rh	N/A	0.0013	**0.005**	0.26	0.0124	**0.002**	0.19
lateraloccipital	T_lh	N/A	0.0005	0.145	0.25	0.0058	**0.041**	0.24	T_rh	N/A	0.0004	0.266	0.24	0.0073	**0.014**	0.23
lateralorbitofrontal	T_lh	N/A	0.0006	0.184	0.13	0.0097	**0.007**	0.12	T_rh	N/A	0.0006	0.150	0.18	0.0118	**0.001**	0.16
lingual	T_lh	N/A	0.0005	**0.040**	0.25	0.0048	**0.013**	0.22	T_rh	N/A	0.0005	**0.043**	0.22	0.0038	0.071	0.18
medialorbitofrontal	T_lh	N/A	0.0004	0.325	0.15	0.0074	**0.036**	0.14	T_rh	N/A	0.0006	0.217	0.12	0.0115	**0.004**	0.11
middletemporal	T_lh	**Linear**	0.0008	0.090	**0.29**	0.0196	**0.000**	**0.27**	T_rh	**Linear**	0.0007	0.190	**0.28**	0.0205	**0.000**	**0.27**
parahippocampal	T_lh	N/A	0.0012	0.068	**0.27**	0.0148	**0.009**	0.24	T_rh	N/A	0.0011	**0.039**	0.20	0.0121	**0.011**	0.17
paracentral	T_lh	N/A	0.0008	0.089	0.14	0.0004	0.926	0.11	T_rh	N/A	0.0009	**0.047**	0.16	0.0017	0.663	0.13
parsopercularis	T_lh	N/A	0.0002	0.428	0.13	0.0049	0.063	0.12	T_rh	N/A	0.0009	**0.015**	0.17	0.0039	0.202	0.11
parsorbitalis	T_lh	N/A	0.0005	0.335	0.11	0.0087	**0.037**	0.10	T_rh	N/A	0.0008	0.077	0.12	0.0024	0.538	0.09
parstriangularis	T_lh	N/A	0.0002	0.510	0.10	0.0039	0.185	0.10	T_rh	N/A	0.0007	0.070	0.10	0.0048	0.152	0.07
pericalcarine	T_lh	N/A	0.0004	0.079	0.13	0.0022	0.268	0.10	T_rh	N/A	0.0002	0.339	0.10	0.0037	**0.048**	0.09
postcentral	T_lh	N/A	0.0006	0.069	0.13	0.0022	0.418	0.10	T_rh	N/A	0.0006	0.070	0.17	0.0061	**0.021**	0.14
posteriorcingulate	T_lh	N/A	0.0012	**0.005**	0.15	0.0057	0.123	0.08	T_rh	N/A	0.0009	**0.026**	0.17	0.0079	**0.023**	0.13
precentral	T_lh	N/A	0.0008	0.070	0.19	0.0009	0.812	0.16	T_rh	N/A	0.0006	0.164	0.17	0.0023	0.522	0.15
precuneus	T_lh	N/A	0.0014	**0.001**	0.19	0.0067	0.060	0.09	T_rh	N/A	0.0011	**0.003**	0.21	0.0082	**0.014**	0.13
rostralanteriorcingulate	T_lh	N/A	-0.0002	0.699	0.18	0.0143	**0.002**	0.18	T_rh	N/A	0.0006	0.318	0.09	0.0067	0.168	0.08
rostralmiddlefrontal	T_lh	N/A	0.0006	0.058	0.19	0.0061	**0.033**	0.16	T_rh	N/A	0.0008	**0.018**	0.14	0.0033	0.240	0.09
superiorfrontal	T_lh	N/A	0.0009	**0.033**	0.19	0.0064	0.064	0.15	T_rh	N/A	0.0008	0.052	0.16	0.0063	0.058	0.12
superiorparietal	T_lh	N/A	0.0012	**0.001**	0.15	0.0007	0.825	0.05	T_rh	N/A	0.0008	**0.035**	0.09	0.0036	0.271	0.05
superiortemporal	T_lh	**Linear**	0.0004	0.289	**0.35**	0.0144	**0.000**	**0.34**	T_rh	**Linear**	0.0006	0.164	**0.29**	0.0177	**0.000**	**0.27**
supramarginal	T_lh	**U**	0.0009	**0.007**	**0.27**	-	-	-	T_rh	N/A	0.0012	**0.004**	0.25	0.0115	**0.002**	0.17
frontalpole	T_lh	N/A	0.0008	0.214	0.10	0.0094	0.093	0.09	T_rh	N/A	0.0010	0.089	0.11	0.0074	0.128	0.08
temporalpole	T_lh	N/A	0.0004	0.678	0.22	0.0320	**0.000**	0.22	T_rh	N/A	0.0021	**0.035**	0.22	0.0313	**0.000**	0.19
transversetemporal	T_lh	N/A	0.0006	0.201	0.21	0.0079	**0.048**	0.20	T_rh	N/A	0.0006	0.277	0.20	0.0128	**0.005**	0.19
insula	T_lh	N/A	0.0005	0.184	0.25	0.0118	**0.000**	0.23	T_rh	**U**	0.0010	**0.033**	**0.33**	-	-	-
