# Supplementary material for: Comparison of neurodegenerative types using different brain MRI analysis metrics in older adults with normal cognition, mild cognitive impairment, and Alzheimer’s dementia
Source: PLoS One. 2019 Aug 1;14(8):e0220739. doi: 10.1371/journal.pone.0220739 (PMC6675320; doi:10.1371/journal.pone.0220739)
Supplement: S6 Table — a coefficient β1 that is for the score2; b p-value from the F-test for the coefficient β1; c coefficient α1 that is for the score of the model w/o score2; d p-value from the coefficient α1; Bold represents significant results. (PDF) [file pone.0220739.s007.pdf]

	Measur	Type	Model w/ score^2			Model w/o score^2			Measur	Type	Model w/ score^2			Model w/o score^2		
			β_1[a]	p-	R^2	α_1[c]	p-	R^2			β_1[a]	p-	R^2	α_1[c]	p-	R^2
bankssts	V_lh	**Linea**	0.8973	0.213	**0.3**	26.8781	**0.000**	**0.3**	V_rh	**Linea**	-	0.975	**0.2**	14.8518	**0.015**	**0.2**
caudalanteriorcingulate	V_lh	N/A	-	0.794	0.05	9.4598	0.130	0.05	V_rh	N/A	-	0.398	0.07	10.8147	0.168	0.06
caudalmiddlefrontal	V_lh	**U**	5.1744	**0.001**	**0.2**	-	-	-	V_rh	**U**	3.376	**0.021**	**0.2**	-	-	-
cuneus	V_lh	N/A	0.3166	0.632	0.21	14.1973	**0.012**	0.21	V_rh	N/A	1.078	0.115	0.24	7.5313	0.193	0.22
entorhinal	V_lh	**Linea**	-	0.846	**0.3**	26.8848	**0.000**	**0.3**	V_rh	N/A	0.990	0.180	**0.2**	24.3475	**0.000**	0.26
fusiform	V_lh	**Linea**	3.8110	0.126	**0.3**	82.0396	**0.000**	**0.3**	V_rh	**Linea**	0.610	0.787	**0.3**	69.1197	**0.000**	**0.3**
inferiorparietal	V_lh	**U**	6.1702	**0.036**	**0.3**	-	-	-	V_rh	**U**	8.366	**0.019**	**0.3**	-	-	-
inferiortemporal	V_lh	**Linea**	3.0446	0.203	**0.3**	84.8123	**0.000**	**0.3**	V_rh	**Linea**	2.248	0.395	**0.4**	123.068	**0.000**	**0.4**
isthmuscingulate	V_lh	N/A	1.5643	**0.017**	0.26	22.3033	**0.000**	0.21	V_rh	**Linea**	0.382	0.526	**0.3**	23.0918	**0.000**	**0.2**
lateraloccipital	V_lh	N/A	5.5163	**0.029**	0.25	31.7232	0.141	0.21	V_rh	**Linea**	2.408	0.336	**0.3**	44.5352	**0.036**	**0.3**
lateralorbitofrontal	V_lh	**Linea**	0.1611	0.893	**0.3**	23.9575	**0.019**	**0.3**	V_rh	N/A	1.441	0.240	0.25	24.0431	**0.021**	0.24
lingual	V_lh	N/A	0.7081	0.630	0.23	33.4942	**0.008**	0.23	V_rh	N/A	1.362	0.319	0.20	20.7358	0.073	0.19
medialorbitofrontal	V_lh	N/A	-	0.710	0.25	14.8154	0.111	0.25	V_rh	N/A	0.622	0.520	0.22	20.9567	**0.011**	0.22
middletemporal	V_lh	**Linea**	1.0838	0.636	**0.4**	130.837	**0.000**	**0.4**	V_rh	**Linea**	1.559	0.585	**0.4**	133.187	**0.000**	**0.4**
parahippocampal	V_lh	**Linea**	0.8651	0.129	**0.3**	19.3297	**0.000**	**0.3**	V_rh	N/A	0.736	0.223	0.19	13.2330	**0.010**	0.18
paracentral	V_lh	N/A	1.0419	0.225	0.26	-3.7228	0.606	0.24	V_rh	N/A	1.877	0.052	**0.3**	-1.0598	0.897	**0.2**
parsopercularis	V_lh	N/A	0.9254	0.392	0.15	17.6385	0.054	0.14	V_rh	N/A	1.059	0.314	0.18	5.0513	0.568	0.17
parsorbitalis	V_lh	N/A	0.1337	0.793	0.06	8.5736	**0.047**	0.06	V_rh	N/A	1.069	0.098	0.13	7.4627	0.172	0.10
parstriangularis	V_lh	N/A	0.8202	0.360	0.05	11.9578	0.114	0.04	V_rh	N/A	1.017	0.395	0.07	10.2610	0.307	0.07
pericalcarine	V_lh	N/A	0.8404	0.148	0.10	5.3009	0.280	0.08	V_rh	N/A	0.487	0.445	0.11	3.2799	0.540	0.10
postcentral	V_lh	N/A	1.0906	0.542	0.23	37.3652	**0.014**	0.23	V_rh	N/A	1.400	0.473	0.24	54.1940	**0.001**	0.23
posteriorcingulate	V_lh	N/A	1.3267	0.110	0.22	18.3156	**0.010**	0.20	V_rh	N/A	1.357	0.108	0.16	16.8608	**0.019**	0.14
precentral	V_lh	N/A	2.5996	0.330	**0.2**	19.2523	0.391	**0.2**	V_rh	N/A	4.188	0.114	0.25	3.9604	0.859	0.23
precuneus	V_lh	N/A	4.2806	**0.047**	0.25	55.3506	**0.003**	0.22	V_rh	**U**	4.155	**0.048**	**0.3**	-	-	-
rostralanteriorcingulat	V_lh	N/A	1.1494	0.148	0.14	14.3777	**0.034**	0.12	V_rh	N/A	-	0.472	0.14	15.2277	**0.018**	0.13
rostralmiddlefrontal	V_lh	**Linea**	3.7940	0.170	**0.3**	81.3110	**0.001**	**0.3**	V_rh	**U**	5.940	**0.040**	**0.3**	-	-	-
superiorfrontal	V_lh	**Linea**	3.8928	0.253	**0.4**	65.5538	**0.024**	**0.3**	V_rh	**Linea**	0.852	0.809	**0.2**	73.4893	**0.014**	**0.2**
superiorparietal	V_lh	N/A	6.1513	**0.019**	0.21	32.9911	0.141	0.16	V_rh	N/A	5.325	0.054	0.18	38.7355	0.100	0.15
superiortemporal	V_lh	**Linea**	1.9032	0.351	**0.4**	69.1500	**0.000**	**0.4**	V_rh	**Linea**	-	0.950	**0.3**	85.9446	**0.000**	**0.3**
supramarginal	V_lh	N/A	4.2681	0.059	**0.2**	55.9890	**0.004**	0.25	V_rh	N/A	1.912	0.409	0.24	70.3635	**0.000**	0.23
frontalpole	V_lh	N/A	0.4119	0.176	0.05	3.1462	0.221	0.03	V_rh	N/A	0.159	0.656	0.06	3.1237	0.299	0.06
temporalpole	V_lh	N/A	-	0.938	0.15	21.0924	**0.002**	0.15	V_rh	N/A	1.330	0.096	0.14	20.3351	**0.003**	0.11
transversetemporal	V_lh	N/A	0.0445	0.899	0.22	7.1733	**0.016**	0.21	V_rh	N/A	0.031	0.902	0.19	6.1538	**0.005**	0.19
insula	V_lh	**Linea**	0.8700	0.445	**0.3**	31.0839	**0.002**	**0.3**	V_rh	**Linea**	0.850	0.573	**0.2**	46.8512	**0.000**	**0.2**
