# Supplementary material for: Comparison of neurodegenerative types using different brain MRI analysis metrics in older adults with normal cognition, mild cognitive impairment, and Alzheimer’s dementia
Source: PLoS One. 2019 Aug 1;14(8):e0220739. doi: 10.1371/journal.pone.0220739 (PMC6675320; doi:10.1371/journal.pone.0220739)
Supplement: S7 Table — a coefficient β1 that is for the score2; b p-value from the F-test for the coefficient β1; c coefficient α1 that is for the score of the model w/o score2; d p-value from the coefficient α1; Bold represents significant results. (PDF) [file pone.0220739.s008.pdf]

	Measure	Type	Model w/ $score^2$			Model w/o $score^2$			Measure	Type	Model w/ $score^2$			Model w/o $score^2$		
			β_1[a]	p-	R^2	α_1[c]	p-	R^2			β_1[a]	p-	R^2	α_1[c]	p-	R^2
bankssts	S_lh	**Linear**	0.1772	0.529	**0.28**	6.2588	**0.009**	**0.28**	S_rh	N/A	-	0.289	**0.29**	1.5828	0.417	**0.28**
caudalanteriorcingulate	S_lh	N/A	-0.1176	0.606	0.14	2.9431	0.125	0.14	S_rh	N/A	-	0.349	0.16	2.5752	0.265	0.16
caudalmiddlefrontal	S_lh	**U**	0.9858	**0.042**	**0.33**	-	-	-	S_rh	N/A	0.2996	0.523	**0.34**	6.8252	0.084	**0.34**
cuneus	S_lh	N/A	-0.0392	0.908	0.20	6.2973	**0.028**	0.20	S_rh	N/A	0.2703	0.413	0.19	2.9921	0.280	0.19
entorhinal	S_lh	N/A	-0.2737	0.086	0.13	-0.3624	0.787	0.10	S_rh	N/A	-	0.295	0.17	-0.7509	0.502	0.16
fusiform	S_lh	N/A	-0.3907	0.558	**0.36**	10.3553	0.066	**0.36**	S_rh	**inverted**	-	**0.020**	**0.35**	-	-	-
inferiorparietal	S_lh	**Linear**	0.3331	0.714	**0.48**	26.3463	**0.001**	**0.48**	S_rh	**Linear**	0.5908	0.563	**0.41**	31.5266	**0.000**	**0.41**
inferiortemporal	S_lh	N/A	-0.5315	0.432	**0.41**	9.6854	0.090	**0.41**	S_rh	**Linear**	-	0.217	**0.46**	20.2320	**0.000**	**0.46**
isthmuscingulate	S_lh	N/A	-0.2123	0.401	0.25	2.5956	0.222	0.25	S_rh	**inverted**	-	**0.046**	**0.35**	-	-	-
lateraloccipital	S_lh	N/A	0.9588	0.283	**0.30**	0.0962	0.990	**0.29**	S_rh	N/A	0.1524	0.867	**0.30**	4.4382	0.559	**0.30**
lateralorbitofrontal	S_lh	N/A	-0.5418	0.228	**0.37**	0.4900	0.897	**0.36**	S_rh	N/A	-	0.756	**0.28**	-0.0375	0.992	**0.28**
lingual	S_lh	N/A	-0.3993	0.525	0.21	11.7982	**0.027**	0.20	S_rh	N/A	-	0.802	0.17	6.0145	0.222	0.17
medialorbitofrontal	S_lh	N/A	-0.6092	0.106	**0.41**	0.8862	0.780	**0.40**	S_rh	N/A	-	0.199	**0.32**	-0.8707	0.748	**0.31**
middletemporal	S_lh	**Linear**	-0.5405	0.359	**0.43**	20.4726	**0.000**	**0.42**	S_rh	**Linear**	-	0.153	**0.45**	19.6437	**0.000**	**0.44**
parahippocampal	S_lh	**Linear**	-0.1253	0.316	**0.41**	3.4033	**0.002**	**0.41**	S_rh	N/A	-	0.477	0.14	2.4216	0.065	0.13
paracentral	S_lh	N/A	-0.0988	0.761	0.18	-2.1735	0.424	0.18	S_rh	N/A	0.1764	0.607	**0.30**	-1.5423	0.591	**0.30**
parsopercularis	S_lh	N/A	0.2632	0.466	0.18	4.4263	0.145	0.18	S_rh	N/A	-	0.803	0.21	1.2520	0.689	0.21
parsorbitalis	S_lh	N/A	-0.0587	0.664	0.16	0.9590	0.397	0.16	S_rh	N/A	0.1003	0.589	0.26	2.7813	0.076	0.25
parstriangularis	S_lh	N/A	0.2516	0.432	0.09	2.9236	0.277	0.08	S_rh	N/A	-	0.696	0.16	1.8893	0.560	0.16
pericalcarine	S_lh	N/A	0.2925	0.433	0.08	1.9574	0.532	0.07	S_rh	N/A	0.2601	0.510	0.12	-1.5000	0.650	0.12
postcentral	S_lh	**Linear**	-1.0595	0.122	**0.33**	13.7126	**0.019**	**0.32**	S_rh	N/A	-	0.218	**0.28**	12.6248	0.057	**0.27**
posteriorcingulate	S_lh	N/A	-0.1491	0.574	**0.28**	3.7959	0.090	**0.27**	S_rh	N/A	-	0.859	0.23	3.4359	0.158	0.23
precentral	S_lh	N/A	-0.6048	0.398	**0.42**	6.3623	0.290	**0.42**	S_rh	N/A	0.1604	0.836	**0.35**	-0.9585	0.882	**0.35**
precuneus	S_lh	N/A	-0.6542	0.351	0.25	12.1294	**0.041**	0.24	S_rh	**Linear**	-	0.540	**0.27**	15.4595	**0.018**	**0.26**
rostralanteriorcingulate	S_lh	N/A	0.3845	0.143	0.20	1.8343	0.407	0.18	S_rh	N/A	-	0.115	0.20	2.8906	0.155	0.17
rostralmiddlefrontal	S_lh	**Linear**	-0.4006	0.695	**0.47**	22.2749	**0.010**	**0.47**	S_rh	**Linear**	-	0.790	**0.48**	22.4118	**0.012**	**0.48**
superiorfrontal	S_lh	N/A	-1.0391	0.311	**0.53**	9.3518	0.278	**0.52**	S_rh	N/A	-	0.117	**0.46**	11.3089	0.178	**0.45**
superiorparietal	S_lh	N/A	-0.7263	0.462	0.19	15.1525	0.069	0.18	S_rh	N/A	-	0.698	**0.26**	8.9250	0.197	**0.26**
superiortemporal	S_lh	N/A	-0.0030	0.996	**0.40**	6.0297	0.203	**0.40**	S_rh	N/A	-	0.084	**0.34**	8.2828	0.088	**0.32**
supramarginal	S_lh	N/A	-0.0446	0.953	**0.32**	8.0866	0.201	**0.32**	S_rh	N/A	-	0.095	0.25	12.6125	**0.044**	0.23
frontalpole	S_lh	N/A	0.0092	0.878	0.02	0.5359	0.283	0.02	S_rh	N/A	-	0.481	0.12	0.1360	0.823	0.12
temporalpole	S_lh	N/A	-0.0594	0.585	0.12	1.0536	0.249	0.12	S_rh	N/A	-	0.640	0.14	0.2232	0.822	0.14
transversetemporal	S_lh	N/A	-0.0388	0.741	0.19	0.8888	0.366	0.19	S_rh	N/A	-	0.351	0.13	0.3581	0.624	0.12
insula	S_lh	N/A	-0.2531	0.420	**0.39**	1.7930	0.496	**0.39**	S_rh	N/A	-	0.179	**0.35**	4.4080	0.218	**0.33**
