# Supplementary material for: Comparison of neurodegenerative types using different brain MRI analysis metrics in older adults with normal cognition, mild cognitive impairment, and Alzheimer’s dementia
Source: PLoS One. 2019 Aug 1;14(8):e0220739. doi: 10.1371/journal.pone.0220739 (PMC6675320; doi:10.1371/journal.pone.0220739)
Supplement: S8 Table — a coefficient β1 that is for the score2; b p-value from the F-test for the coefficient β1; c coefficient α1 that is for the score of the model w/o score2; d p-value from the coefficient α1; Bold represents significant results. (PDF) [file pone.0220739.s009.pdf]

	Measure	Type	Model w/ score^2			Model w/o score^2			Measure	Type	Model w/ score^2			Model w/o score^2		
			β_1[a]	p-	R^2	α_1[c]	p-	R^2			β_1[a]	p-	R^2	α_1[c]	p-	R^2
bankssts	LGI_lh	N/A	-0.0001	0.831	0.19	0.0127	**0.000**	0.19	LGI_rh	N/A	-	0.744	0.17	0.0106	**0.001**	0.16
caudalanteriorcingulate	LGI_lh	N/A	-0.0001	0.396	0.07	-0.0017	0.230	0.06	LGI_rh	N/A	-	0.425	0.14	-	0.774	0.13
caudalmiddlefrontal	LGI_lh	N/A	-0.0003	0.393	0.08	0.0010	0.693	0.07	LGI_rh	N/A	0.0000	0.983	0.10	0.0033	0.139	0.10
cuneus	LGI_lh	N/A	0.0002	0.654	0.04	0.0029	0.318	0.04	LGI_rh	N/A	0.0002	0.652	0.01	0.0002	0.944	0.01
entorhinal	LGI_lh	N/A	0.0001	0.814	0.09	-0.0017	0.410	0.09	LGI_rh	N/A	-	0.550	0.09	0.0014	0.508	0.09
fusiform	LGI_lh	N/A	0.0000	0.861	0.12	0.0016	0.287	0.12	LGI_rh	N/A	0.0001	0.746	0.14	0.0020	0.151	0.14
inferiorparietal	LGI_lh	N/A	-0.0001	0.817	0.07	0.0026	0.167	0.07	LGI_rh	N/A	0.0001	0.548	0.14	0.0041	**0.046**	0.14
inferiortemporal	LGI_lh	N/A	-0.0001	0.760	0.02	0.0021	0.213	0.02	LGI_rh	N/A	0.0000	0.889	0.06	0.0019	0.274	0.06
isthmuscingulate	LGI_lh	N/A	0.0000	0.998	0.12	0.0034	0.217	0.12	LGI_rh	N/A	0.0005	0.171	0.11	0.0027	0.378	0.09
lateraloccipital	LGI_lh	N/A	0.0000	0.865	0.05	0.0016	0.385	0.05	LGI_rh	N/A	0.0002	0.463	0.05	0.0024	0.171	0.05
lateralorbitofrontal	LGI_lh	N/A	0.0000	0.912	0.10	0.0019	0.309	0.10	LGI_rh	N/A	-	**0.025**	0.25	0.0026	0.115	0.21
lingual	LGI_lh	N/A	0.0003	0.298	0.07	0.0029	0.187	0.06	LGI_rh	N/A	0.0002	0.394	0.08	0.0008	0.715	0.07
medialorbitofrontal	LGI_lh	N/A	-0.0001	0.536	0.06	-0.0020	0.153	0.05	LGI_rh	N/A	-	**0.026**	0.11	-	0.603	0.06
middletemporal	LGI_lh	N/A	-0.0005	0.190	0.13	0.0097	**0.002**	0.11	LGI_rh	N/A	-	0.328	0.12	0.0073	**0.005**	0.11
parahippocampal	LGI_lh	N/A	0.0000	0.997	0.21	-0.0001	0.965	0.21	LGI_rh	N/A	0.0000	0.933	0.15	0.0010	0.619	0.15
paracentral	LGI_lh	N/A	-0.0003	0.283	0.02	0.0005	0.796	0.01	LGI_rh	N/A	-	0.507	0.03	-	0.560	0.02
parsopercularis	LGI_lh	N/A	-0.0001	0.796	0.11	0.0083	**0.049**	0.11	LGI_rh	N/A	-	0.216	0.12	0.0107	**0.011**	0.11
parsorbitalis	LGI_lh	N/A	-0.0003	0.387	0.16	0.0060	0.063	0.15	LGI_rh	N/A	-	0.215	0.14	0.0036	0.244	0.13
parstriangularis	LGI_lh	N/A	0.0000	0.985	0.12	0.0060	0.099	0.12	LGI_rh	N/A	0.0001	0.865	0.07	0.0052	0.216	0.07
pericalcarine	LGI_lh	N/A	0.0002	0.532	0.04	0.0025	0.347	0.04	LGI_rh	N/A	0.0003	0.318	0.05	-	0.982	0.04
postcentral	LGI_lh	N/A	-0.0004	0.190	0.17	0.0044	0.072	0.15	LGI_rh	N/A	-	0.578	0.21	0.0058	**0.011**	0.21
posteriorcingulate	LGI_lh	N/A	-0.0004	0.176	0.06	0.0015	0.533	0.04	LGI_rh	N/A	0.0000	0.985	0.06	0.0013	0.518	0.06
precentral	LGI_lh	N/A	-0.0003	0.339	0.14	0.0046	0.053	0.13	LGI_rh	N/A	-	0.457	0.17	0.0057	**0.011**	0.17
precuneus	LGI_lh	N/A	-0.0002	0.579	0.09	0.0035	0.198	0.08	LGI_rh	N/A	0.0001	0.699	0.03	0.0007	0.792	0.03
rostralanteriorcingulate	LGI_lh	N/A	-0.0001	0.603	0.04	-0.0020	0.138	0.04	LGI_rh	N/A	-	0.102	0.10	-	0.580	0.07
rostralmiddlefrontal	LGI_lh	N/A	-0.0001	0.800	0.09	0.0018	0.368	0.09	LGI_rh	N/A	0.0000	0.921	0.13	0.0019	0.300	0.13
superiorfrontal	LGI_lh	N/A	0.0000	0.932	0.01	-0.0011	0.400	0.01	LGI_rh	N/A	-	0.426	0.06	-	0.755	0.05
superiorparietal	LGI_lh	N/A	-0.0001	0.760	0.09	0.0018	0.284	0.09	LGI_rh	N/A	-	0.377	0.03	0.0012	0.537	0.02
superiortemporal	LGI_lh	N/A	-0.0007	0.138	0.17	0.0115	**0.003**	0.15	LGI_rh	N/A	-	0.075	0.23	0.0112	**0.001**	0.20
supramarginal	LGI_lh	N/A	-0.0001	0.664	0.08	0.0035	0.227	0.08	LGI_rh	N/A	-	0.367	0.16	0.0061	**0.018**	0.15
frontalpole	LGI_lh	N/A	-0.0001	0.564	0.09	-0.0023	0.144	0.08	LGI_rh	N/A	-	0.416	0.04	-	0.513	0.04
temporalpole	LGI_lh	N/A	0.0003	0.215	0.04	-0.0009	0.655	0.02	LGI_rh	N/A	-	0.547	0.08	0.0010	0.519	0.08
transversetemporal	LGI_lh	N/A	-0.0008	0.142	0.16	0.0110	**0.018**	0.14	LGI_rh	N/A	-	0.084	0.24	0.0144	**0.001**	0.21
insula	LGI_lh	N/A	-0.0004	0.399	0.17	0.0093	**0.022**	0.16	LGI_rh	N/A	-	0.422	0.16	0.0116	**0.005**	0.15
